# Sparse Granger Causality Analysis Model Based on Sensors Correlation for Emotion Recognition Classification in Electroencephalography

**DOI:** 10.3389/fncom.2021.684373

**Published:** 2021-07-29

**Authors:** Dongwei Chen, Rui Miao, Zhaoyong Deng, Na Han, Chunjian Deng

**Affiliations:** ^1^Zhuhai People's Hospital (Zhuhai Hospital Affiliated With Jinan University), Zhuhai, China; ^2^Faculty of Information Technology, Macau University of Science and Technology, Avenida Wai Long, Taipa, China; ^3^University of Electronic Science and Technology of China, Chengdu, China; ^4^School of Electronic Information Engineering, University of Electronic Science and Technology of China, Zhongshan, China; ^5^School of Business, Beijing Institute of Technology, Zhuhai, China

**Keywords:** granger causality analysis, EEG sensors, LASSO, SC-SGA, L1/2-based sparse granger causality analysis, L2 norm logistic regression

## Abstract

In recent years, affective computing based on electroencephalogram (EEG) data has attracted increased attention. As a classic EEG feature extraction model, Granger causality analysis has been widely used in emotion classification models, which construct a brain network by calculating the causal relationships between EEG sensors and select the key EEG features. Traditional EEG Granger causality analysis uses the *L*_2_ norm to extract features from the data, and so the results are susceptible to EEG artifacts. Recently, several researchers have proposed Granger causality analysis models based on the least absolute shrinkage and selection operator (LASSO) and the *L*_1/2_ norm to solve this problem. However, the conventional sparse Granger causality analysis model assumes that the connections between each sensor have the same prior probability. This paper shows that if the correlation between the EEG data from each sensor can be added to the Granger causality network as prior knowledge, the EEG feature selection ability and emotional classification ability of the sparse Granger causality model can be enhanced. Based on this idea, we propose a new emotional computing model, named the sparse Granger causality analysis model based on sensor correlation (SC-SGA). SC-SGA integrates the correlation between sensors as prior knowledge into the Granger causality analysis based on the *L*_1/2_ norm framework for feature extraction, and uses *L*_2_ norm logistic regression as the emotional classification algorithm. We report the results of experiments using two real EEG emotion datasets. These results demonstrate that the emotion classification accuracy of the SC-SGA model is better than that of existing models by 2.46–21.81%.

## 1. Introduction

Emotions are an important part of decision cognition and interpersonal interaction (Oatley et al., 2006; Izard, 2013), and research in many fields is attempting to recognize human emotions through computer systems, such as emotional computing, neurology, and psychology (Catanzarite and Greenburg, 1979; Picard, 1999). In the field of human interaction, in particular, emotional computing would enable machines to perceive the emotional state of the human brain, allowing them to learn more about people through human–computer interaction (Cauchard et al., 2016; Zhou, 2018). At present, research methods for studying emotion recognition are mainly divided into two categories: the first category is based on non-physiological signals, such as speech, body posture, and facial expression; the second category is based on physiological signals, such as electrocardiogram and electroencephalogram (EEG) data (Picard, 2000, 2003; Tao and Tan, 2005). EEG signals are obtained directly from the cerebral cortex, and thus directly reflect changes in human emotions (Larsen, 2011; Dan et al., 2013). Therefore, in recent years, EEG emotion recognition technology has become increasingly popular (Bos et al., 2006; Lin et al., 2010; Atkinson and Campos, 2016; Song et al., 2018).

Researchers have proposed many advanced EEG analysis methods, such as identifying subtypes of mental disorders from the functional connection patterns of resting state EEG data; improving EEG decoding through cluster-based multitasking feature learning; and early Alzheimer's diagnosis based on resting state EEG topological network analysis (Moore and DeNero, 2011; Wang et al., 2011; Liu et al., 2012; Zhang et al., 2012; Zhou et al., 2012; Zhu et al., 2014; Suk et al., 2015). Among them, feature extraction and sensor causality analysis are hot topics of research. Granger causality analysis is an important feature extraction method based on sentiment calculation, and has been widely used by researchers (Dongwei et al., 2013; Immordino-Yang and Singh, 2013; Zhang et al., 2017). For example, Zhang et al. used the Granger causality analysis model to construct an effective brain connection network on Database for Emotion Analysis Using Physiological Signals (DEAP) emotional EEG data to study how emotion affects the pattern of effective connection (Zhang et al., 2017); Coito et al. used the Granger causality model to study whether the EEG phase of patients with left temporal lobe epilepsy and right temporal lobe epilepsy exhibited changes in directional functional connectivity (Coito et al., 2016). However, clinical and neuroscience applications will inevitably produce outliers or artifacts when collecting data (Blankertz et al., 2007). These can cause the quality of EEG signals to deteriorate and produce problems with noise. In particular, EEG signals are often contaminated by abnormal values when blinking or head movements form a trajectory. The original Granger causality analysis uses the *L*_2_ norm loss function, the squared nature of which tends to exaggerate outliers, and retains all of the data. This can lead to erroneous analysis results (Xu et al., 2007, 2010a; Li et al., 2015; Bore et al., 2018, 2019). Therefore, due to the sparse connectivity of the brain network, researchers proposed Granger causality analysis models based on the least absolute shrinkage and selection operator (LASSO) to solve the noise problem (Valdés-Sosa et al., 2005; Marinazzo et al., 2008; Shaw and Routray, 2018). However, the *L*_1/2_ regularizer is more sparse and robust than LASSO (Xu et al., 2010b; Zong-Ben et al., 2012; Li et al., 2017). Thus, Granger causality analysis based on the *L*_1/2_ norm has been developed, and experiments have proved that this obtains better solutions (Bore et al., 2020).

**Figure 1:**
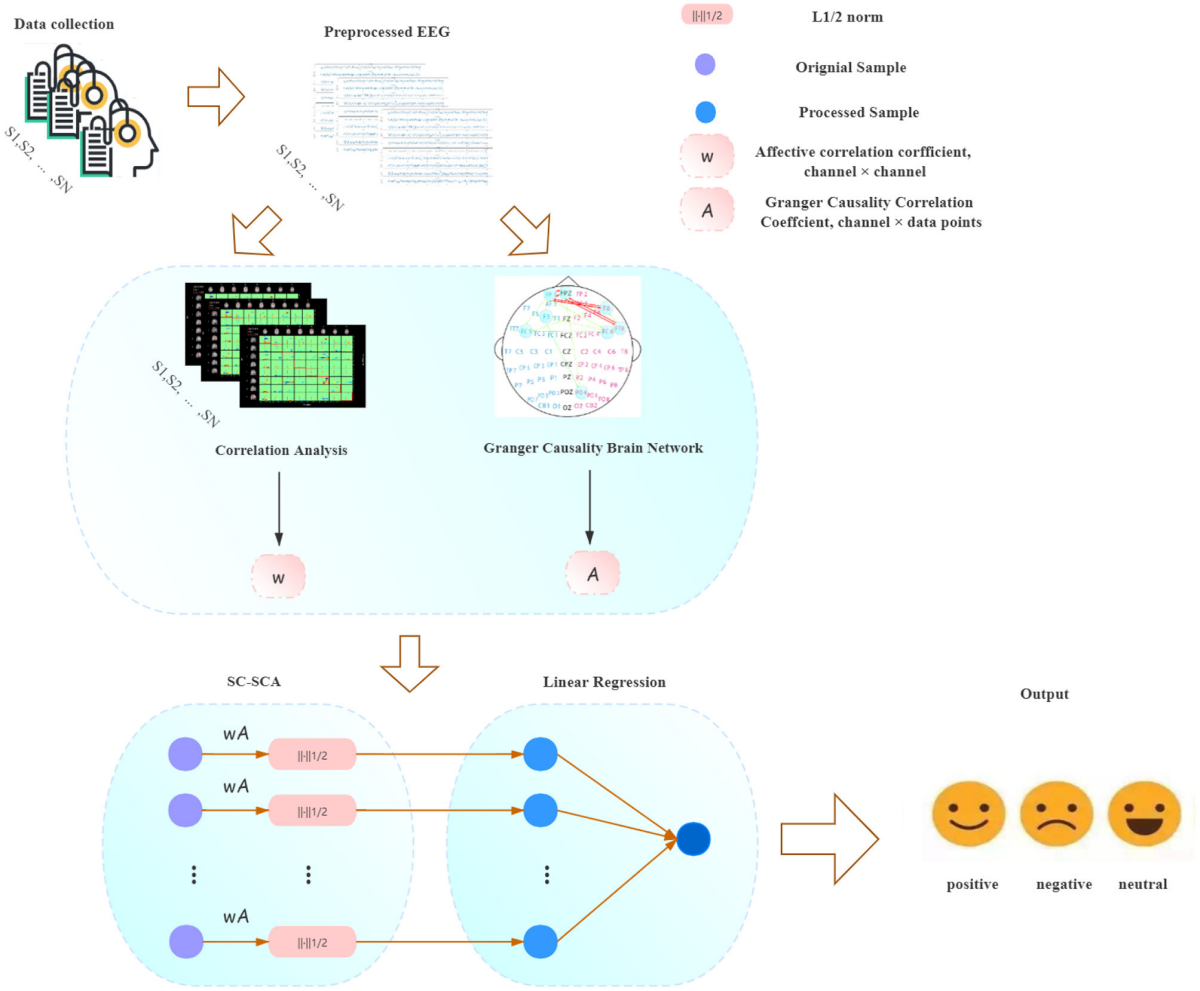
Experimental process of the model proposed in the paper.

The purpose of the existing sparse Granger causality analysis model based on LASSO or *L*_1/2_ regularization is to establish a sparse brain network relationship matrix, retain the data between EEG sensors with high causality, and remove data with weak causality. Hence, effectively calculating the causality weights between EEG sensors has become a key issue in sparse Granger causality analysis. The existing sparse Granger model uses the multivariate autoregressive (MVAR) model to establish the weight matrix of the EEG sensor causality relationship (Geweke, 1982; Seth, 2010; Hu et al., 2015). MVAR reflects the direct causality relationship between each sensor. This method assumes that each EEG sensor has the same prior knowledge (that is, the correlation between the various sensors is consistent). However, based on known EEG data, researchers can use statistical methods to pre-calculate the correlation between each EEG channel. We believe that if the correlation between EEG channels could be integrated into the sparse Granger model as prior knowledge, the causality relationship between the various sensors in the existing sparse Granger causality model would be enhanced, thereby improving the feature selection ability of the model. Based on this idea, the present paper proposes a Granger causality network model based on sparse sensor correlation, and combines a sparse logistic regression classification algorithm based on *L*_2_ regularization. This sparse Granger causality analysis model based on sensor correlation (SC-SGA) uses the Pearson similarity coefficient to calculate the degree of similarity between sensors. SC-SGA integrates this similarity degree as a weight into a sparse Granger causality model based on the *L*_1/2_ regularizer for feature extraction, and finally uses a sparse logistic regression algorithm based on *L*_2_ regularization for emotion recognition, as shown in Figure 1.

In this study, experiments were conducted on two real datasets. The experimental results show that, compared with the existing models, the SC-SGA model achieves better recognition of different emotions. We believe that the SC-SGA model is a good complement to the classification model based on sparse Granger causality analysis, and that the method and results presented in this article will be very useful in future research.

## 2. Materials and Methods

### 2.1. Materials

**Figure 2:**
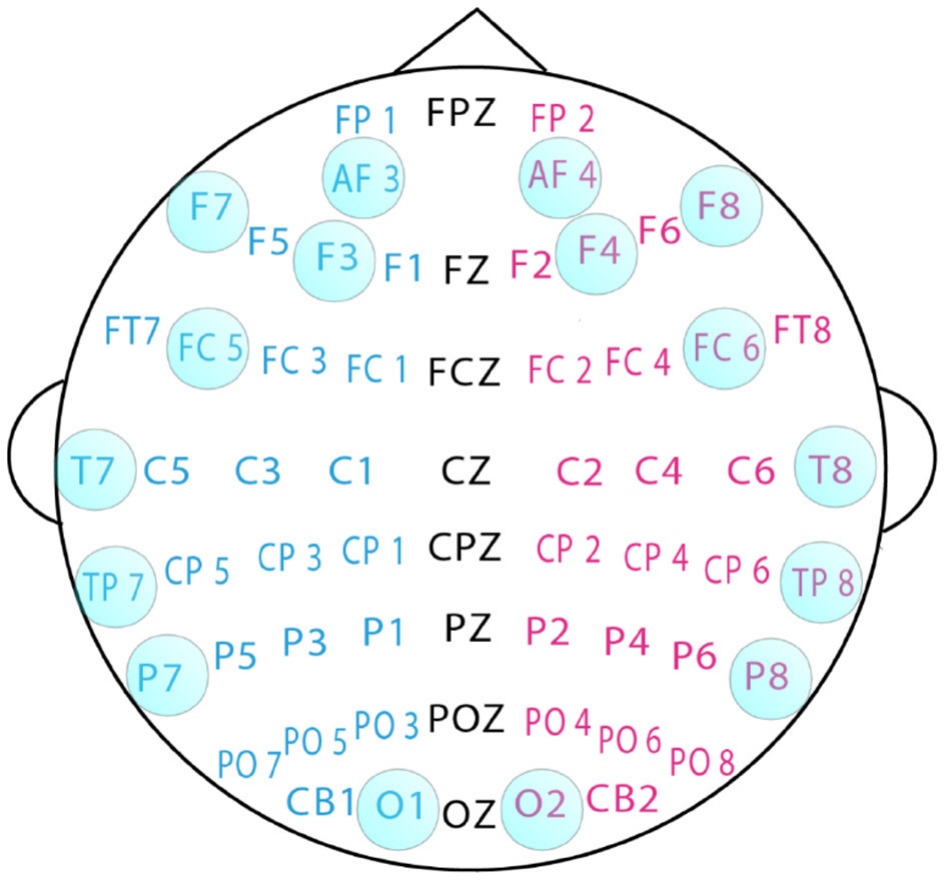
Sixteen channels used in the experiments.

Sixteen channels were selected for experiments related to emotional states. The channel selection is shown in Figure 2.

#### 2.1.1. SEED Dataset

The SJTU(Shanghai Jiao Tong University) Emotion EEG Dataset (SEED) is a collection of EEG datasets provided by the BCMI(Brain-like Computing & Machine Inteligence) laboratory (Duan et al., 2013). SEED uses film fragments as emotion-inducing materials and includes three categories of emotion: positive, neutral, and negative. The details of the film clips used in the experiments are listed in Table 1. A total of 15 subjects (seven males, eight females, mean age 23.27 years, standard deviation 2.37 years) participated in the SEED experiments, all of whom had normal visual, auditory, and emotional states. In the experiments, 15 movie clips were played. These movie clips were all from Chinese movies. The 15 movie clips were of three types, with five clips of each type. Each clip was played for about 4 min. In each experiment, movie clips of different emotional states were watched by the participants. As the subject was watching the movie, EEG signals were recorded through an electrode cap at a sampling frequency of 1,000 Hz. The experiments used the international 10–20 system and a 62-channel electrode cap. Each volunteer participated in three experiments, and each experiment was separated by about 1 week. Therefore, after screening, a total of 660 data samples had been obtained. To obtain a preprocessed EEG dataset, 200 Hz down-sampling and a bandpass frequency filter from 0 to 75 Hz were applied. Each dimension of SEED is described in Table 2. For more information on this dataset, please refer to the website http://bcmi.sjtu.edu.cn/~seed/index.html.

**Table 1:** Details of the film clips used in the SEED dataset.

**No**.	**Emotion label**	**Film clip sources**
1	negative	Tangshan Earthquake
2	negative	Back to 1942
3	positive	Lost in Thailand
4	positive	Flirting Scholar
5	positive	Just Another Pandora's Box
6	neutral	World Heritage in China

**Table 2:** SEED dataset.

**Array name**	**Array shape**	**Array contents**
data	660 × 62 × 185 × 5	*sample*/*trial* × *channel* × *data* × *band*
labels	660 × 1	*sample*/*trial* × *labels*

a) Gamma band dataset: The SEED EEG dataset contains five EEG bands. The main frequency range of the five bands is 1–50 Hz. The frequency range of gamma brain waves is 31–50 Hz. Previous studies have shown that the gamma band generally occurs in pathological conditions, such as epilepsy, or under external stimuli. Additionally, it is often used for multimodal analysis in experiments. Therefore, we use the gamma brain waves for experimental analysis. The gamma brain wave frequency band of the SEED dataset contains 660 samples.

b) Combined band dataset: To verify the performance of our model, we also examine the use of all frequency bands of the EEG dataset. The EEG signals were decomposed into five frequency bands according to the EEG rhythm, comprising delta (1–3 Hz), theta (4–7 Hz), alpha (8–13 Hz), beta (14–30 Hz), and gamma (31–50 Hz) bands. These five frequency band signals were combined to form a new combined frequency band dataset. Therefore, two EEG datasets representing different frequency bands were obtained. Finally, four feature processing models were tested and verified using the above datasets: Original (dataset not processed), LASSO, least absolute *L*_*p*_ (0 < p <1) penalized solution (LAPPS), and SC-SGA. In the experiments, the 660 samples were randomly assigned to a mutually exclusive training set (80%) and a verification set (20%).

(1) $$\begin{array}{l} \{\begin{array}{c} W_{1}\left(t\right)=\sum_{i=1}^{s}a_{11}\left(i\right)W_{1}\left(t-i\right)+\sum_{i=1}^{s}a_{21}\left(i\right)W_{2}\left(t-i\right)+\ldots +\sum_{i=1}^{s}a_{m1}\left(i\right)W_{m}\left(t-i\right)+\varepsilon_{1}\left(t\right),var\left(\varepsilon_{1}\left(t\right)\right)=\sum 1\\ W_{2}\left(t\right)=\sum_{i=1}^{s}a_{12}\left(i\right)W_{1}\left(t-i\right)+\sum_{i=1}^{s}a_{22}\left(i\right)W_{2}\left(t-i\right)+\ldots +\sum_{i=1}^{s}a_{m2}\left(i\right)W_{m}\left(t-i\right)+\varepsilon_{2}\left(t\right),var\left(\varepsilon_{2}\left(t\right)\right)=\sum 2\\ \vdots \\ W_{m}\left(t\right)=\sum_{i=1}^{s}a_{1m}\left(i\right)W_{1}\left(t-i\right)+\sum_{i=1}^{s}a_{2m}\left(i\right)W_{2}\left(t-i\right)+\ldots +\sum_{i=1}^{s}a_{mm}\left(i\right)W_{m}\left(t-i\right)+\varepsilon_{m}\left(t\right),var\left(\varepsilon_{m}\left(t\right)\right)=\sum m \end{array}. \end{array}$$

#### 2.1.2. DEAP Dataset

The DEAP dataset (Koelstra et al., 2011) can be found at http://www.eecs.qmul.ac.uk/mmv/datasets/deap/. It includes 32-channel EEG signals and peripheral physiological signals such as GSR(galvanic skin response) signals, EOG(electro-oculogram) signals, EMG(electromyography) signals, PPG(photoplethysmograph) signals, temperature, and status. All data have been down-sampled to 128 Hz, whereby the EEG signal data became a 60 s test signal and a 3 s baseline. A zero-phase bandpass filter of 4–45 Hz was applied. In this study, the 32-channel EEG data were divided into two classes according to their arousal status: positive (more than 6) and negative (less than 4).

**Figure 3:**
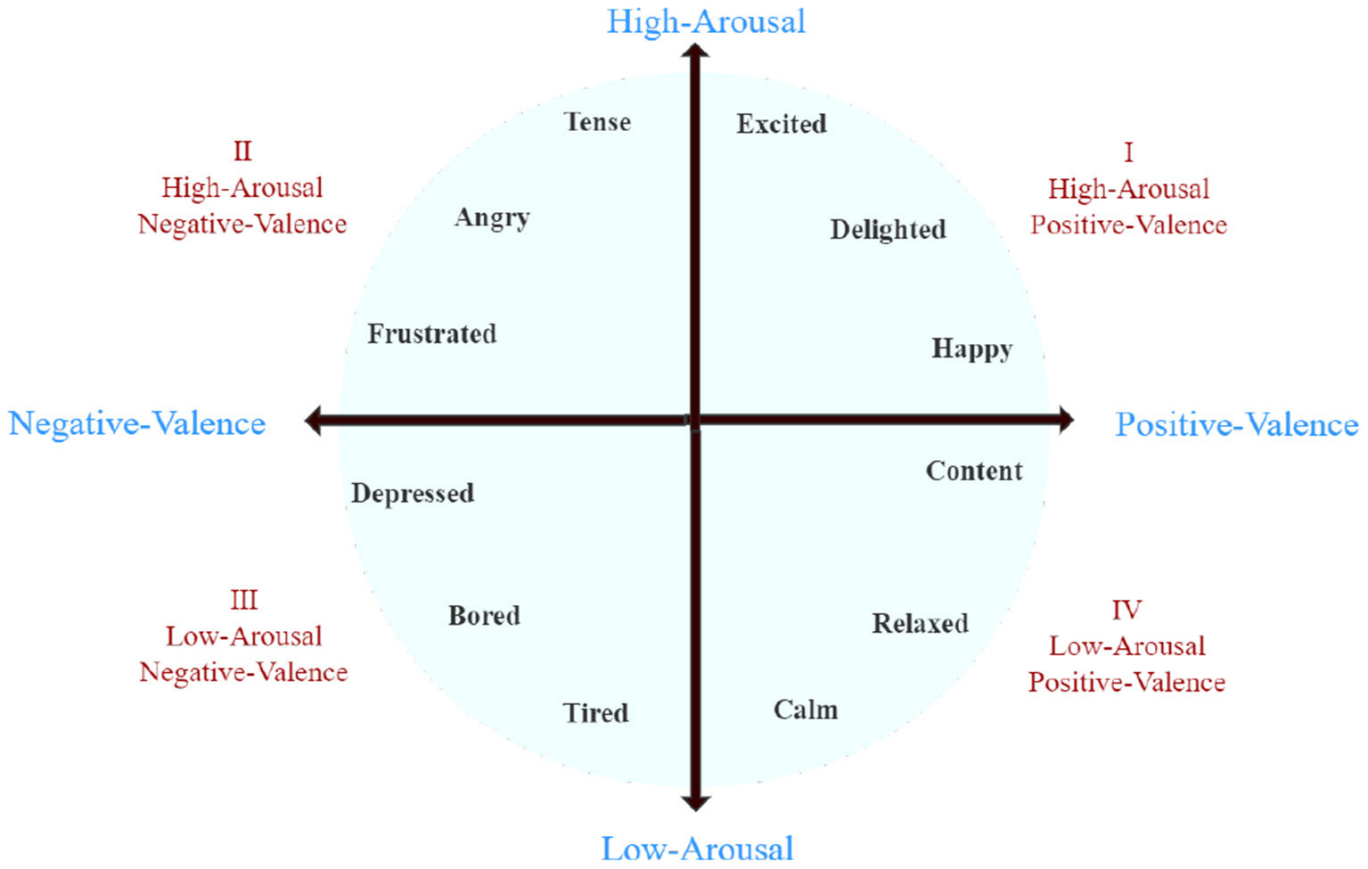
Two-dimensional emotional model.

The DEAP dataset consists of two parts. The first part contains the ratings from an online self-assessment in which 120 1-min extracts of music videos were rated by 14–16 volunteers based on arousal, valence, and dominance. The second part includes the participant ratings, physiological recordings, and facial videos from an experiment in which 32 volunteers watched a subset of 40 of the above music videos. The EEG and physiological signals were recorded and each participant rated the videos as above. For 22 participants, frontal face videos were also recorded. At the end of each video, the participants were required to fill out a self-assessment form of their arousal, ranging from inactive (1) to active (9), their valence, ranging from unpleasant (1) to pleasant (9), and their dominance feelings, ranging from helpless and weak (1) to empowered (9). Figure 3 shows the two-dimensional emotional model of the DEAP dataset. Each dimension of DEAP is described in Table 3. In experiments with the DEAP dataset, we only used data from combined frequency bands.

**Table 3:** DEAP dataset.

**Array name**	**Array shape**	**Array contents**
data	40 × 40 × 8064	*video*/*trial* × *channel* × *data*
labels	40 × 4	*video*/*trial* × *labels*

#### 2.1.3. Cross-Validation

To ensure the accuracy of the results, a 5-fold cross-validation method was used in all the experiments. Five-fold cross-validation first divides all the data into five sub-samples. One of the sub-samples is selected as the test set, and the other four samples are used for training. This process is repeated five times, and the average and its error range are calculated. In addition to 5-fold cross-validation, all experiments described in this paper were performed 100 times, allowing the average and error statistics to be obtained.

### 2.2. Methods

The sparsity of connections in brain networks has been proved by researchers (Genç et al., 2018). Many unnecessary connections will occur when researchers construct causality brain networks. If these connections are directly involved in the analysis and calculation, there will be an increased computational complexity and an enhanced likelihood of overfitting. Therefore, when constructing causality brain networks, sparse regularizers such as the *L*_1_ and *L*_2_ norm can be used. Adding sparse regularizers effectively extracts the important features of the network and reduces the time complexity of the network. In this way, the goal of improving accuracy while reducing the operational requirements can be achieved (Bore et al., 2018, 2020).

#### 2.2.1. *L*_2_ Granger Analysis

Granger analysis is based on an MVAR model. This form of analysis allows researchers to estimate the relationship between multiple sets of time series data. Therefore, the accuracy with which the MVAR parameters are calculated determines the reliability of the final relationship, which ultimately affects the accuracy of the Granger analysis correlation network. There are multiple strategies for estimating the parameters of MVAR models. If we assume there are *m* stationary stochastic processes with *W*_*i*_(*t*) ∈ *R* time domain observations such that *i* = 1, 2, …, *m*; *t* − 1, 2, …, *T*, we obtain Equation (1), where *s* is the maximum number of lagged observations that are added to the model and *a*_*ij*_(*i* = 1, 2…, *m*; *j* = 1, 2, …, *m*) is the vector of coefficients that defines the effect of the activity of *W*_*i*_(*t*) on *W*_*j*_(*t*). Moreover, $\underset{k}{\sum }\left(k=1,2,\ldots ,m\right)$ is the variance of residuals between the expected *W*_*k*_ and the predicted *Ŵ*_*k*_ in the corresponding processes. Suppose that:

(2) $$\begin{array}{l} X_{k}={\left[a_{1k}\left(1\right),\ldots ,a_{1k}\left(s\right),\ldots ,a_{mk}\left(1\right),\ldots ,a_{mk}\left(s\right)\right]}^{T} \end{array}$$

are the multivariate autoregressive coefficients, with *m* being the number of time series and *y*_*k*_ = [*W*_*k*_(*s*+1), *W*_*k*_(*s*+2), …, *W*_*k*_(*n*)] being the *n* − *s* elements to be predicted for *W*_*k*_, where *n* denotes the length of the signal. Now, we define the design matrix *A* ∈ R^(*n*−*s*)×(*m*×*s*)^ as:

(3) $$\begin{array}{l} A=\left[Z_{1}\;Z_{2}\;\ldots \;Z_{k}\;\ldots \;Z_{m}\right] \end{array}$$

In this case:

(4) $$\begin{array}{l} Z_{i}=\left[\begin{array}{cccc} W_{i}\left(s\right) & W_{i}\left(s-1\right) & \ldots & W_{i}\left(1\right)\\ W_{i}\left(s+1\right) & W_{i}\left(s\right) & \ldots & W_{i}\left(2\right)\\ \vdots & \vdots & \vdots & \vdots \\ W_{i}\left(n-1\right) & W_{i}\left(n-2\right) & \ldots & W_{i}\left(n-s\right) \end{array}\right] \end{array}$$

Consequently, we find the solution for Equation (1) with the objective term defined in the *L*_2_ norm space (*L*_2_ norm loss function) as:

(5) $$\begin{array}{l} \underset{x_{k}}{argmin}f_{k}\left(X_{k}\right)=∥y_{k}-AX_{k}{∥}_{2}^{2} \end{array}$$

Here, ∥·∥_2_ denotes the *L*_2_ norm of a vector and “argmin” indicates that the best solution minimizes the objective function *f*_*k*_(*X*_*k*_). By taking the derivative of Equation (5) with respect to *X*_*k*_ under the condition (*df*_*k*_)/(*dX*_*k*_) = 0, we obtain the following formulation:

(6) $$\begin{array}{l} 2A^{T}AX_{k}-2A^{T}y_{k}=0 \end{array}$$

The MVAR coefficients for process *W*_*k*_ are given by:

(7) $$X_{k}=\{\begin{array}{c} {\left(A^{T}A\right)}^{-1}A^{T}y_{k}\text{ if }A^{T}A\text{ is nonsingular}\\ {\left(A^{T}A\right)}^{+}A^{T}y_{k}\text{ if }A^{T}A\text{ is singular} \end{array}.$$

where (*A*^*T*^*A*)−1 is the inverse operation of *A*^*T*^*A* and (*A*^*T*^*A*)+ indicates the pseudo-inverse of *A*^*T*^*A* (Watkins, 2004).

#### 2.2.2. LASSO Granger Analysis

Because the neurons in the brain are sparsely connected, retaining all the information between the sensors may cause erroneous analysis results due to noise. Therefore, Granger analysis based on LASSO has been developed. LASSO uses the *L*_1_ norm, and adding the *L*_1_ norm to Granger analysis can reduce some coefficients to zero, thus obtaining sparse results. Based on Equation (5), we can write:

(8) $$\begin{array}{l} LASSO\text{\_}GA=argmin\left\{∥y-AX{∥}_{2}^{2}+\lambda ∥X{∥}_{1}\right\} \end{array}$$

where λ ≥ 0 is a regularization parameter. This formula is a classic convex optimization problem that can be solved using a greedy algorithm.

#### 2.2.3. LAPPS Granger Analysis

Recently, researchers have discovered that the *L*_1/2_ norm is a more sparse and robust regularizer than the *L*_1_ norm. Therefore, a Granger analysis model based on the *L*_1/2_ norm has been proposed. The model estimates the MVAR parameters using LAPPS. The model is theoretically sparser than that given by LASSO. The ability to eliminate noise and artifacts is also stronger. The Granger analysis model based on the *L*_1/2_ norm can be written as:

(9) $$\begin{array}{l} LAPPS\text{\_}GA=\underset{X}{min}\left\{\frac{1}{\eta }∥AX-y{∥}_{1}+∥X{∥}_{P}^{P}\right\} \end{array}$$

where the fitting error is measured in the *L*_1_ norm space and $L_{P}\left(p=\frac{1}{2}\right)$ norm regularization is imposed on the coefficients, while η > 0 is the regularization parameter. The alternating direction method of multipliers (ADMM) framework can be used to solve this problem.

#### 2.2.4. Proposed LAPPS Granger Analysis Based on Sensor Correlation

In the EEG emotion recognition model, the key factor in improving the final experimental result is feature extraction. Finding EEG data that are related to emotion is the core problem of feature extraction. However, in the previous sparse Granger analysis model, each sensor has the same prior knowledge. This means that the final feature extraction result is only related to the value of the EEG signal, and does not necessarily correspond to the emotional state. If we can quantify the correlation between each EEG sensor and emotion (Chen et al., 2020), and use this as a weight in the sparse Granger analysis model, the model's feature selection ability would be improved, further improving the model's classification ability. Under this idea, based on existing research, we propose a sparse Granger analysis model based on sensor correlation and the *L*_1/2_ norm. The model can be written as follows:

(10) $$\begin{array}{l} LALF\text{\_}GA=\underset{X}{min}\left\{∥y-EAX{∥}_{1}+∥X{∥}_{P}^{P}\right\} \end{array}$$

where *L*_*P*_ $\left(p=\frac{1}{2}\right)$ norm regularization is imposed on the coefficients. *E* represents the sensor correlation, which can also be approximated as the weight of emotion. We hope to retain as much relationship information related to emotion as possible. The formula for calculating *E* is as follows:

(11) $$\begin{array}{l} E=\left[T_{1}\;T_{2}\;\ldots \;T_{m}\right] \end{array}$$

where T is the number of time series. In this case:

(12) $$\begin{array}{l} T_{i}=\left[\begin{array}{cccc} M_{11} & M_{12} & \ldots & M_{i1}\\ M_{21} & M_{22} & \ldots & M_{i2}\\ \vdots & \vdots & \vdots & \vdots \\ M_{1j} & M_{2j} & \ldots & M_{ij} \end{array}\right] \end{array}$$

where *i* and *j* represent the number of sensors. For *M*_*i*_, we have:

(13) $$\begin{array}{l} M_{ij}=\frac{Cov\left(M_{i},M_{j}\right)}{\sqrt{Var\left[M_{i}\right]Var\left[M_{j}\right]}} \end{array}$$

where *Cov*(*M*_*i*_, *M*_*j*_) represents the covariance of the *i*-th and *j*-th sensors, *Var*[*M*_*i*_] represents the variance of the *i*-th sensor, and *Var*[*M*_*j*_] represents the variance of the *j*-th sensor.

## 3. Classification Methods

### 3.1. Logistic Regression Model

In this study, logistic regression is used as the classification model. The probability formula of logistic regression is as follows Wright (1995):

(14) $$\begin{array}{l} p\left(y=1|x\right)=\frac{1}{1+e^{-wx}} \end{array}$$

### 3.2. *L*_2_ Sparse Regularizer

Considering the high-dimensional characteristics of EEG data, a logistic regression model based on the *L*_2_ regularizer is used. The formula for the *L*_2_ regularizer is as follows Cortes et al. (2012):

(15) $$\begin{array}{l} \Phi \left(w\right)=\sum w^{2} \end{array}$$

### 3.3. Support Vector Machine Model

As well as the logistic regression model based on sparsity, a support vector machine (SVM) model is used for classification and comparison. The SVM model is a two-classification technique. Its basic model is a linear classifier of the largest interval defined in the feature space, which is the most amenable to the perceptual machine (Adnan et al., 2020; Li et al., 2020; Wang and Chen, 2020). The SVM model also includes kernel techniques, which makes it an effective nonlinear classifier. The learning strategy for the SVM involves maximizing the interval and formalizing a convex quadratic programming problem, which is equivalent to the problem of minimizing the regular closed loss function. The learning algorithm of the SVM model is the optimization algorithm for solving convex quadratic programming problems (Scholkopf and Smola, 2018).

## 4. Experimental Results

A series of experiments were conducted using two real datasets and four sparse Granger causal models, namely the Original-Granger causal model, LASSO-Granger causal model, LAPPS model, and the proposed SC-SGA model. The classifier for each model was built using the SVM method, logistic regression method, and ridge regression method. Confusion matrices are used to compare the results between the various models. These matrices summarize the prediction results of classification models in machine learning. The records in the dataset are summarized in matrix form according to the real category and the classification criteria predicted by the classification model. The rows of the matrix represent the true values, and the columns represent the predicted values. The computational accuracy of the proposed model is used as a measure of quality, where the accuracy is defined as the ratio of the number of samples correctly classified by the classifier to the total number of samples in the test dataset. However, accuracy is not always an effective metric for performance evaluation, especially if the numbers of samples with different labels are not exactly equal. Therefore, we also analyze the precision and recall for further comparison of the three two-classifier models. Here, precision refers to the proportion of all predicted true positives in positive classes, and recall refers to the proportion of positives found in all positive classes. All experiments used 5-fold cross-validation to ensure the stability of the proposed model.

### 4.1. SEED Dataset

As shown in Table 4 and Supplementary Table I, the experimental results using the gamma band show that the SC-SGA model proposed in this paper has obvious advantages over the other models. In terms of neutral emotion, the experimental results using the SVM method give a precision of 84.70% for the proposed model, which is 22.70, 10.23, and 2.48% higher than the Original, LASSO-GA, and LAPPS models, respectively. The recall of our proposed model is 87.03%, which is 13.22, 9.25, and 0.98% higher than that of the Original, LASSO-GA, and LAPPS models, respectively. In the experimental results using the logistic regression method, the precision of our proposed model is 88.89%, which is 16.80 13.10 and 3.18% higher than with the Original, LASSO-GA, and LAPPS models, respectively. The recall of our proposed model is 79.12%, which is 5.31, 3.36, and 0.86% higher than that of the Original, LASSO-GA, and LAPPS models, respectively. In the experimental results using the ridge regression method, the precision of our proposed model is 85.99%, which is 15.78, 9.39, and 0.62% higher than that of the Original, LASSO-GA, and LAPPS models, respectively. The recall of our proposed model is 84.70%, which is 12.96, 4.70, and 1.37% higher than with the Original, LASSO-GA, and LAPPS models, respectively.

**Table 4:** Precision and recall results for ridge regression using the gamma band of the SEED dataset.

**Feature extraction model**	**Neutral**		**Positive**		**Negative**	
	**Precision**	**Recall**	**Precision**	**Recall**	**Precision**	**Recall**
Original	0.7021 ± 0.0310	0.7174 ± 0.0295	0.5909 ± 0.0278	0.6667 ± 0.0320	0.6829 ± 0.0286	0.5957 ± 0.0304
LASSO-GA	0.7660 ± 0.0221	0.8000 ± 0.0248	0.8333 ± 0.0214	0.8489 ± 0.0213	0.8649 ± 0.0201	0.7619 ± 0.0193
LAPPS	0.8537 ± 0.0132	0.8333 ± 0.0154	0.8400 ± 0.0182	0.8733 ± 0.0173	0.8780 ± 0.0148	0.8000 ± 0.0146
SC-SGA	0.8599 ± 0.0105	0.8470 ± 0.0110	0.8821 ± 0.0092	0.8975 ± 0.0103	0.8979 ± 0.0114	0.8360 ± 0.0108

In terms of negative emotion, we obtain a similar conclusion. In the experimental results using the ridge regression method, the precision of the proposed model is 89.79%, some 21.50, 3.30, and 1.99% higher than the Original, LASSO-GA, and LAPPS models, respectively. The recall of SC-SGA is 83.60%, which is 24.03, 7.41, and 3.60% higher than that of the Original, LASSO-GA, and LAPPS models, respectively. In classifying positive emotion, the proposed method achieves the best experimental results. Using ridge regression, the precision of our proposed model is 88.21%, which is 29.12, 4.88, and 4.21% higher than the Original, LASSO-GA, and LAPPS models, respectively. The recall of our proposed model is 89.75%, which is 23.08, 4.86, and 2.42% higher than that of the Original, LASSO-GA, and LAPPS models, respectively. With the ridge regression method, the precision of our proposed model is 86.49%, which is 20.58, 4.67, and 1.88% higher than the Original, LASSO-GA, and LAPPS models, respectively. (See the Supplementary Materials for the *L*_1_ and SVM results.)

We also experimented with the combined band of the SEED dataset. The three classification methods were used with the four processing models, and the results are consistent with those for the gamma band. The following analysis considers the results obtained by ridge regression (for the SVM and *L*_1_ results, see the Supplementary Materials).

The results in Table 5 indicate that, when ridge regression is used as the classification method, the SC-SGA model achieves the best precision and recall of the four emotional classification models. For neutral emotion, the precision of our proposed model is 85.98%, which is 11.98, 2.88, and 1.60% higher than the Original, LASSO-GA, and LAPPS models, respectively. The recall of our proposed model is 86.12%, which is 7.40, 3.90, and 1.74% higher than that of the Original, LASSO-GA, and LAPPS models, respectively. In terms of positive emotion, the precision of our proposed model is 86.18%, which is 5.23, 2.44, and 2.05% higher than Original, LASSO-GA, and LAPPS, respectively. The recall of our proposed model is 87.88%, approximately 0.70, 8.33, and 0.71% higher than when using the Original, LASSO-GA, and LAPPS models, respectively. Regarding negative emotion, the precision of our proposed model is 83.15%, which is 10.65, 10.07, and 8.15% higher than that of the Original, LASSO-GA, and LAPPS models, respectively. The recall of our proposed model is 87.56%, which is 15.82, 3.19, and 7.20% higher than with the Original, LASSO-GA, and LAPPS models, respectively.

**Table 5:** Precision and recall results for ridge regression using the combined band of the SEED dataset.

**Feature extraction model**	**Neutral**		**Positive**		**Negative**	
	**Precision**	**Recall**	**Precision**	**Recall**	**Precision**	**Recall**
Original	0.7400 ± 0.0280	0.7872 ± 0.0301	0.8095 ± 0.0293	0.7718 ± 0.0284	0.7250 ± 0.0312	0.7174 ± 0.0205
LASSO-GA	0.8310 ± 0.0243	0.8222 ± 0.0212	0.8374 ± 0.0250	0.7955 ± 0.0201	0.7308 ± 0.0224	0.8437 ± 0.0198
LAPPS	0.8438 ± 0.0156	0.8438 ± 0.0127	0.8413 ± 0.0149	0.8717 ± 0.0186	0.7500 ± 0.0190	0.8036 ± 0.0198
SC-SGA	0.8598 ± 0.0124	0.8612 ± 0.0106	0.8618 ± 0.0096	0.8788 ± 0.0101	0.8315 ± 0.0090	0.8756 ± 0.0104

**Figure 4:**
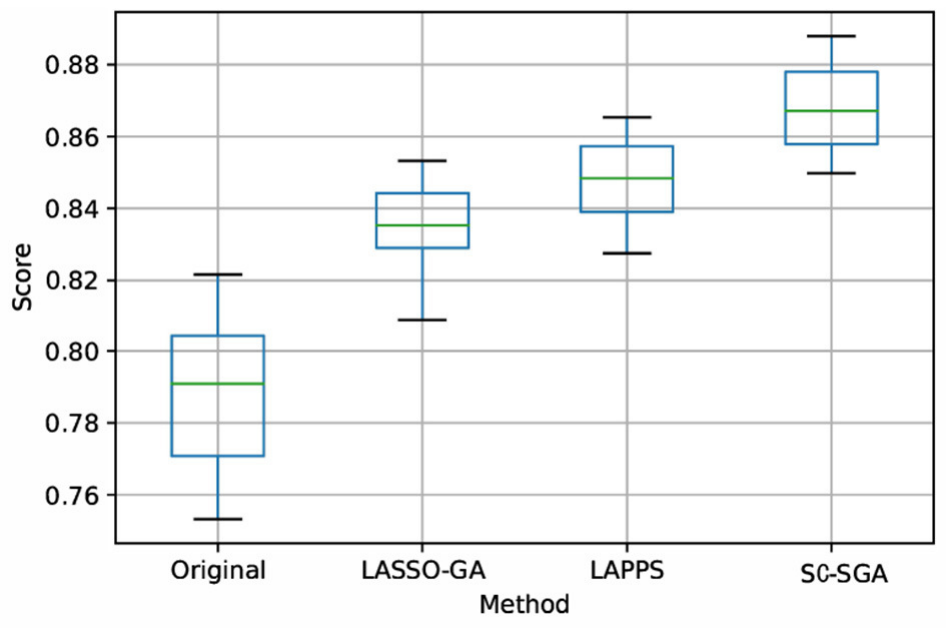
Box plot obtained using ridge regression with the four models for the combined band of the SEED dataset. Score represents the accuracy rate.

Figures 4, 5 in the Supplementary Materials and Figure 4 are box plots obtained from experiments using the four processing models with SVM, *L*_1_, and ridge regression classifiers for the combined band of the SEED dataset. Clearly, the accuracy of SC-SGA is better than that of the other three classification models and the results are more robust, with fewer fluctuations, which produces a better effect. In particular, the ridge regression method produces smaller fluctuations than the other two classifiers.

**Figure 5:**
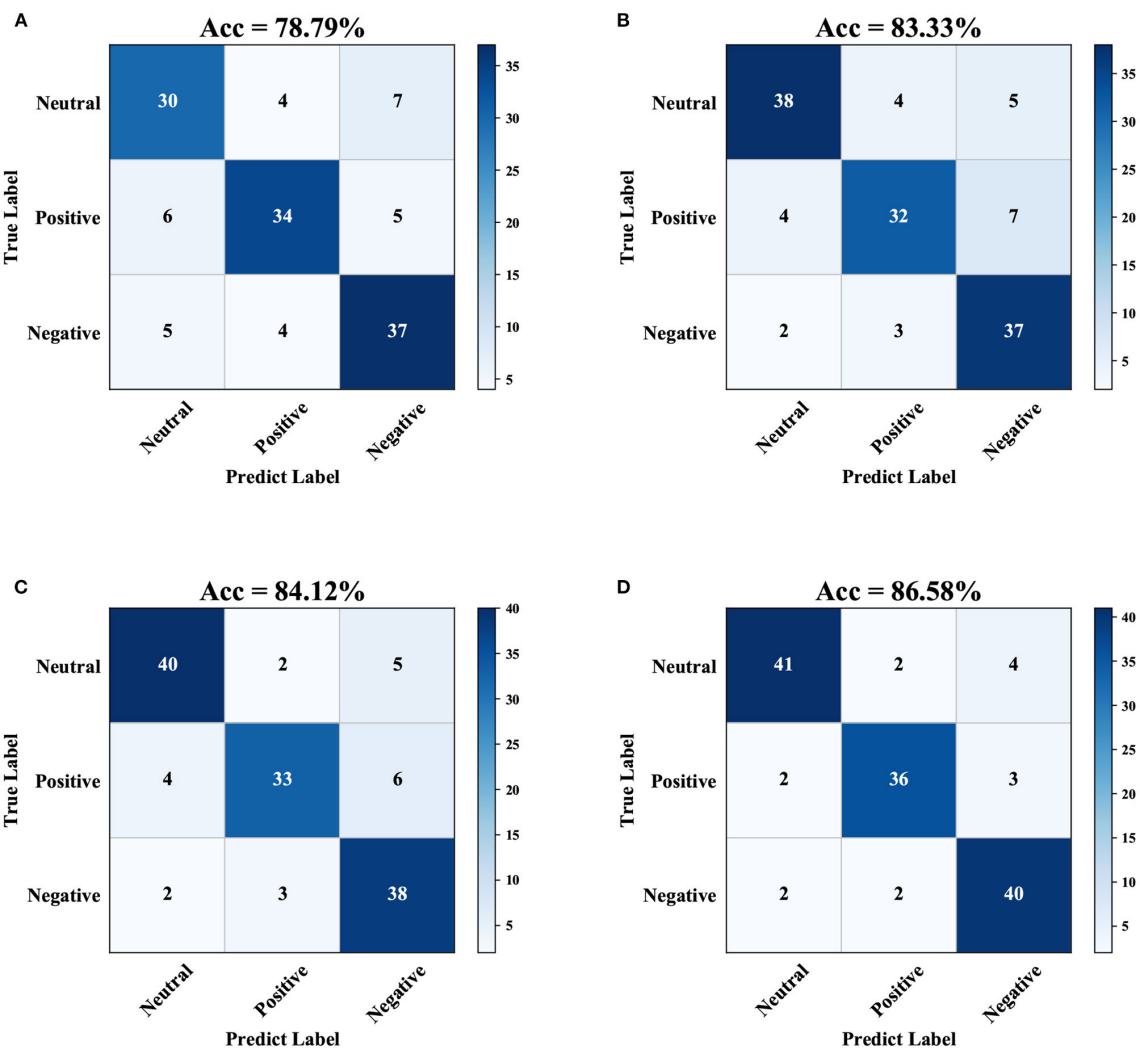
Confusion matrices obtained using ridge regression under the processing of the four models for the combined band of the SEED dataset. The darker the cell color, the more samples allocated in the interval. **(A)** Original dataset, **(B)** Using LASSO-G, **(C)** Using LAPPS, and **(D)** Using SC-SGA.

Figures 5A–D show the confusion matrices obtained by using the four processing models under the ridge regression classification method for the combined band of the SEED dataset. These data show that, among the four processing models, the SC-SGA model achieves the highest accuracy of 86.58%, which is 7.79, 3.25, and 2.46% higher than the accuracy of the Original, LASSO-GA, and LAPPS models, respectively. Among the three emotions, the SC-SGA processing model gives the fewest wrongly classified samples (15 samples), while the Original, LASSO-GA, and LAPPS models produce 31, 25, and 22 classification errors, respectively. The results show that SC-SGA is better than other models in dealing with the SEED dataset. To further validate the model proposed in this article, we now analyze the results using the DEAP dataset.

### 4.2. DEAP Dataset

The experimental results using the DEAP dataset are analyzed in Table 6. Similar to the results with the SEED dataset, the SC-SGA model produces the best effect. The detailed results using SVM and *L*_1_ are given in the Supplementary Materials, and the results using ridge regression are analyzed below.

**Table 6:** Precision and recall results for ridge regression using the DEAP dataset.

**Feature extraction model**	**Positive**		**Negative**	
	**Precision**	**Recall**	**Precision**	**Recall**
Original	0.7132 ± 0.0350	0.6846 ± 0.0332	0.7297 ± 0.0314	0.7049 ± 0.0321
LASSO-GA	0.7219 ± 0.0243	0.7106 ± 0.0251	0.7496 ± 0.0220	0.7513 ± 0.0201
LAPPS	0.7699 ± 0.0190	0.7488 ± 0.0143	0.7806 ± 0.0158	0.7843 ± 0.0176
SC-SGA	0.8016 ± 0.0120	0.7931 ± 0.0123	0.8102 ± 0.0092	0.8267 ± 0.0102

In terms of positive emotion, the precision of the proposed model is 80.16%, which is 8.84, 7.79, and 3.17% higher than that of the Original, LASSO-GA, and LAPPS models, respectively. The recall of the proposed model is 79.31%, which is 10.85, 8.25, and 4.43% higher than with the Original, LASSO-GA, and LAPPS models, respectively. For negative emotion, the precision of the proposed model is 81.02%, which is 8.05, 6.06, and 2.96% higher than that of the Original, LASSO-GA, and LAPPS models, respectively. The recall of the proposed model is 82.67%, which is 12.18, 7.54, and 4.24% higher than when using the Original, LASSO-GA, and LAPPS models, respectively.

The above results indicate that LASSO-GA, LAPPS, and SC-SGA have improved to a certain extent. However, the improvement effect of the SC-SGA model is better than that of the existing LASSO-GA and LAPPS models. This demonstrates that SC-SGA provides support for superior emotion classification.

**Figure 6:**
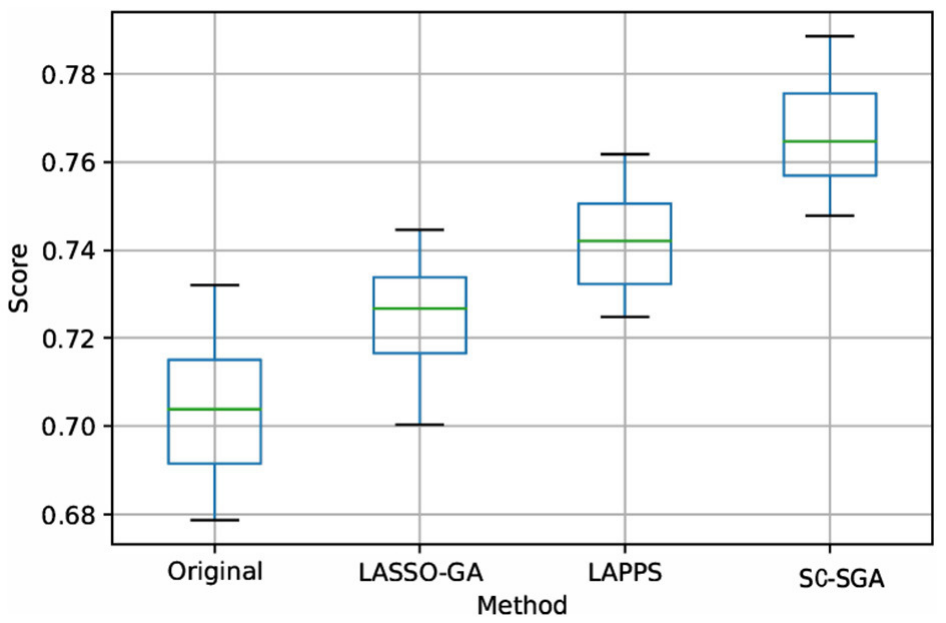
Box plot obtained using ridge regression with the four models for the DEAP dataset. Score represents the accuracy rate.

Figure 5, 7 in the Supplementary Materials and Figure 6 are box plots obtained from experiments using the four processing models with SVM, *L*_1_, and ridge regression applied to the DEAP dataset. Clearly, the accuracy of SC-SGA is better than that of the other three classification models. As far as stability is concerned, SC-SGA produces smaller fluctuations and is more stable.

It can therefore be concluded that the SC-SGA model is superior to existing models in the experiments conducted on these two real datasets. This proves that the SC-SGA model is better able to solve the problem of false connections caused by abnormal values.

## 5. Discussion

In emotional computing, feature selection is the key to improving model performance. A classic algorithm for EEG feature extraction is the brain network based on Granger causality analysis. However, the inevitable abnormal values in EEG measurements can lead to false connections. Therefore, researchers have developed Granger causality analysis models based on LASSO and causality analysis based on the *L*_1/2_ norm for denoising. However, in the construction of the brain network based on Granger causality analysis, the characteristic EEG data are retained by analyzing the causality relationships between the EEG sensors. Thus, accurately analyzing the causality relationship between sensors and assigning appropriate weights have become the focus of research. The existing sparse Granger causality model does not consider the use of prior knowledge. However, based on known EEG sensor timing signals, researchers can directly analyze the degree of correlation between sensors. We believe that if the timing signals of two sensors are more closely related, they are more likely to have a causality relationship.

On the basis of this idea, we proposed the model described in this article, using the known sensor correlations as prior knowledge to enhance the causality construction ability of the existing sparse Granger model. Based on the existing literature, we selected 16 emotion-related sensor channels and used the *L*_1/2_ norm to remove artifacts in the data while retaining emotion-related information (Zheng and Lu, 2015; Zheng et al., 2017; Chen et al., 2020). Next, we calculated the similarity between sensors. We assumed that the similarity between these sensors was related to the sensor correlation, which means that the similarity degree could be used as a correction to enhance the ability of the model to distinguish different emotional states. From a neurobiological perspective, the cortical electrodes record the total discharge of neurons, and the discharge state of different emotions must be different. Therefore, the similarity between sensors should be used as the *a* priori weight for sensor causal analysis. The experimental results strongly support our hypothesis.

Although we have proved that the similarity between sensors can enhance the feature selection ability of the Granger causality model, the experimental constructions in this paper are based only on cerebral cortex signals and do not trace the EEG signals. Therefore, in future work, we will further improve this model so that it can be applied to the data after EEG traceability. This will enable further study of the relationship between the sensor similarity and sensor causality.

## 6. Conclusion

The experimental results presented in this paper show that, compared with existing models, the proposed SC-SGA model has better emotion recognition capabilities and stability. We believe that this model provides an excellent supplement to classification models based on sparse Granger causality analysis. We hope that the proposed model will provide new ideas for the development of sparse Granger causality models, thus promoting the clinical application of the auxiliary diagnosis of affective disorders in the brain science industry.

## Data Availability Statement

The original contributions presented in the study are included in the article/Supplementary Material, further inquiries can be directed to the corresponding author/s.

## Ethics Statement

We used two public datasets, called SEED and DEAP. The studies involving human participants were reviewed and approved by the DEAP dataset team and the SJTU Emotion EEG Dataset team. The patients/participants provided their written informed consent to participate in this study.

## Author Contributions

ZD, RM, and DC proposed the method to write this paper, ZD conducted experiments, CD and NH read the manuscript and modified it. All authors contributed to the manuscript and approved the submitted version.

## Conflict of Interest

The authors declare that the research was conducted in the absence of any commercial or financial relationships that could be construed as a potential conflict of interest.

## Publisher's Note

All claims expressed in this article are solely those of the authors and do not necessarily represent those of their affiliated organizations, or those of the publisher, the editors and the reviewers. Any product that may be evaluated in this article, or claim that may be made by its manufacturer, is not guaranteed or endorsed by the publisher.

## References

[B1] Adnan R. M. Liang Z. Heddam S. Zounemat-Kermani M. Kisi O. Li B. (2020). Least square support vector machine and multivariate adaptive regression splines for streamflow prediction in mountainous basin using hydro-meteorological data as inputs. J. Hydrol. 586, 124371. 10.1016/j.jhydrol.2019.124371

[B2] Atkinson J. Campos D. (2016). Improving bci-based emotion recognition by combining eeg feature selection and kernel classifiers. Exp. Syst. Appl. 47, 35–41. 10.1016/j.eswa.2015.10.049

[B3] Blankertz B. Dornhege G. Krauledat M. Müller K.-R. Curio G. (2007). The non-invasive berlin brain-computer interface: fast acquisition of effective performance in untrained subjects. NeuroImage 37, 539–550. 10.1016/j.neuroimage.2007.01.051 17475513

[B4] Bore J. C. Ayedh W. M. A. Li P. Yao D. Xu P. (2019). Sparse autoregressive modeling via the least absolute lp-norm penalized solution. IEEE Access 7, 40959–40968. 10.1109/ACCESS.2019.2908189

[B5] Bore J. C. Li P. Harmah D. J. Li F. Yao D. Xu P. (2020). Directed eeg neural network analysis by lapps (p ≤ 1) penalized sparse granger approach. Neural Netw. 124, 213–222. 10.1016/j.neunet.2020.01.022 32018159

[B6] Bore J. C. Yi C. Li P. Li F. Harmah D. J. Si Y.. (2018). Sparse eeg source localization using lapps: Least absolute l-p (0 < p <1) penalized solution. IEEE Trans. Biomed. Eng. 66, 1927–1939. 10.1109/TBME.2018.2881092 30442597

[B7] Bos D. O. (2006). Eeg-based emotion recognition. Influence Vis. Aud. Stimuli 56, 1–17.

[B8] Catanzarite V. A. Greenburg A. G. (1979). * neurologist*: computer program for diagnosis in neurology, in Proceedings of the Annual Symposium on Computer Application in Medical Care, American Medical Informatics Association, 64.

[B9] Cauchard J. R. Zhai K. Y. Spadafora M. Landay J. A. (2016). Emotion encoding in human-drone interaction, in 2016 11th ACM/IEEE International Conference on Human-Robot Interaction (HRI). (IEEE), 263–270.

[B10] Chen D.-W. Miao R. Deng Z.-Y. Lu Y.-Y. Liang Y. Huang L. (2020). Sparse logistic regression with l1/2 penalty for emotion recognition in electroencephalography classification. Front. Neuroinform. 14:9. 10.3389/fninf.2020.00029 32848688 PMC7427509

[B11] Coito A. Genetti M. Pittau F. Iannotti G. R. Thomschewski A. Höller Y.. (2016). Altered directed functional connectivity in temporal lobe epilepsy in the absence of interictal spikes: a high density eeg study. Epilepsia 57, 402–411. 10.1111/epi.13308 26890734

[B12] Cortes C. Mohri M. Rostamizadeh A. (2012). l_2_ regularization for learning kernels. *arXiv preprint arXiv*:1205.2653.

[B13] Dan Z. Xifeng Z. Qiangang G. (2013). An identification system based on portable eeg acquisition equipment, in textit2013 Third International Conference on Intelligent System Design and Engineering Applications. (IEEE), 281–284.

[B14] Dongwei C. Fang W. Zhen W. Haifang L. Junjie C. (2013). Eeg-based emotion recognition with brain network using independent components analysis and granger causality., in 2013 International Conference on Computer Medical Applications (ICCMA). (IEEE), 1–6.

[B15] Duan R.-N. Zhu J.-Y. Lu B.-L. (2013). Differential entropy feature for EEG-based emotion classification, in 6th International IEEE/EMBS Conference on Neural Engineering (NER). (IEEE), 81–84.

[B16] Genç E. Fraenz C. Schlüter C. Friedrich P. Hossiep R. Voelkle M. C.. (2018). Diffusion markers of dendritic density and arborization in gray matter predict differences in intelligence. Nat. Commun. 9, 1–11. 10.1038/s41467-018-04268-8 29765024 PMC5954098

[B17] Geweke J. (1982). Measurement of linear dependence and feedback between multiple time series. J. Am. Stat. Assoc. 77, 304–313. 10.1080/01621459.1982.10477803

[B18] Hu S. Wang H. Zhang J. Kong W. Cao Y. Kozma R. (2015). Comparison analysis: Granger causality and new causality and their applications to motor imagery. IEEE Trans. Neural Netw. Learn. Syst. 27, 1429–1444. 10.1109/TNNLS.2015.2441137 26099149

[B19] Immordino-Yang M. H. Singh V. (2013). Hippocampal contributions to the processing of social emotions. Hum. Brain Mapp. 34, 945–955. 10.1002/hbm.21485 22012639 PMC3340463

[B20] Izard C. E. (2013). Human Emotions. Springer Science Business Media.

[B21] Koelstra S. Muhl C. Soleymani M. Lee J.-S. Yazdani A. Ebrahimi T.. (2011). Deap: A database for emotion analysis; using physiological signals. IEEE Trans. Affect. Comput. 3, 18–31. 10.1109/T-AFFC.2011.15

[B22] Larsen E. A. (2011). Classification of EEG Signals in a Brain-Computer Interface System. Master's thesis, Institutt for datateknikk og informasjonsvitenskap.

[B23] Li L.-L. Zhao X. Tseng M.-L. Tan R. R. (2020). Short-term wind power forecasting based on support vector machine with improved dragonfly algorithm. J. Cleaner Product. 242, 118447. 10.1016/j.jclepro.2019.118447

[B24] Li P. Huang X. Li F. Wang X. Zhou W. Liu H.. (2017). Robust granger analysis in lp norm space for directed eeg network analysis. IEEE Trans. Neural Syst. Rehabil. Eng. 25, 1959–1969. 10.1109/TNSRE.2017.2711264 28600253

[B25] Li P. Wang X. Li F. Zhang R. Ma T. Peng Y.. (2015). Autoregressive model in the lp norm space for eeg analysis. J. Neurosci. Methods 240, 170–178. 10.1016/j.jneumeth.2014.11.007 25448380

[B26] Lin Y.-P. Wang C.-H. Jung T.-P. Wu T.-L. Jeng S.-K. Duann J.-R.. (2010). Eeg-based emotion recognition in music listening. IEEE Trans. Biomed. Eng. 57, 1798–1806. 10.1109/TBME.2010.2048568 20442037

[B27] Liu J. Ji S. Ye J. (2012). Multi-task feature learning via efficient l2,1-norm minimization. arXiv e-prints, page arXiv:1205.2631.

[B28] Marinazzo D. Pellicoro M. Stramaglia S. (2008). Kernel method for nonlinear granger causality. Phys. Rev. Lett. 100, 144103. 10.1103/PhysRevLett.100.144103 18518037

[B29] Moore R. DeNero J. (2011). L1 and l2 regularization for multiclass hinge loss models, in Symposium on Machine Learning in Speech and Language Processing.

[B30] Oatley K. Keltner D. Jenkins J. M. (2006). Understanding Emotions. Blackwell Publishing.

[B31] Picard R. W. (1999). Affective computing for HCI, in HCI (1). (Citeseer), 829–833.

[B32] Picard R. W. (2000). Affective Computing. MIT Press.

[B33] Picard R. W. (2003). Affective computing: challenges. Int. J. Hum. Comput. Stud. 59, 55–64. 10.1016/S1071-5819(03)00052-1

[B34] Scholkopf B. Smola A. J. (2018). Learning with kernels: support vector machines, regularization, optimization, and beyond, in Adaptive Computation and Machine Learning Series.

[B35] Seth A. K. (2010). A matlab toolbox for granger causal connectivity analysis. J. Neurosci. Methods 186, 262–273. 10.1016/j.jneumeth.2009.11.020 19961876

[B36] Shaw L. Routray A. (2018). A new framework to infer intra- and inter-brain sparse connectivity estimation for eeg source information flow. IEEE Sens. J. 18, 10134–10144. 10.1109/JSEN.2018.2875377

[B37] Song T. Zheng W. Song P. Cui Z. (2018). Eeg emotion recognition using dynamical graph convolutional neural networks, in IEEE Transactions on Affective Computing.

[B38] Suk H.-I. Wee C.-Y. Lee S.-W. Shen D. (2015). Supervised discriminative group sparse representation for mild cognitive impairment diagnosis. Neuroinformatics 13, 277–295. 10.1007/s12021-014-9241-6 25501275 PMC4469635

[B39] Tao J. Tan T. (2005). Affective computing: a review, in International Conference on Affective Computing and Intelligent Interaction. (Springer), 981–995.

[B40] Valdés-Sosa P. A. Sánchez-Bornot J. M. Lage-Castellanos A. Vega-Hernández M. Bosch-Bayard J. Melie-García L.. (2005). Estimating brain functional connectivity with sparse multivariate autoregression. Philos. Trans. R. Soc. B Biol. Sci. 360, 969–981. 10.1098/rstb.2005.1654 16087441 PMC1854937

[B41] Wang H. Nie F. Huang H. Risacher S. Saykin A. J. Shen L.. (2011). Identifying ad-sensitive and cognition-relevant imaging biomarkers via joint classification and regression, in International Conference on Medical Image Computing and Computer-Assisted Intervention. (Springer), 115–123 10.1007/978-3-642-23626-6_15 PMC3201708 22003691

[B42] Wang M. Chen H. (2020). Chaotic multi-swarm whale optimizer boosted support vector machine for medical diagnosis. Appl. Soft Comput. 88, 105946. 10.1016/j.asoc.2019.105946

[B43] Watkins D. S. (2004). Fundamentals of Matrix Computations, Vol. 64. John Wiley Sons.

[B44] Wright R. E. (1995). Logistic regression, in Reading and Understanding Multivariate Statistics, eds Grimm L. G. Yarnold P. R. (American Psychological Association), 217–244.

[B45] Xu P. Tian Y. Chen H. Yao D. (2007). Lp norm iterative sparse solution for eeg source localization. IEEE Trans. Biomed. Eng. 54, 400–409. 10.1109/TBME.2006.886640 17355051

[B46] Xu P. Tian Y. Lei X. Yao D. (2010a). Neuroelectric source imaging using 3sco: a space coding algorithm based on particle swarm optimization and *l*_0_ norm constraint. NeuroImage 51, 183–205. 10.1016/j.neuroimage.2010.01.106 20139015

[B47] Xu Z. Zhang H. Wang Y. Chang X. Liang Y. (2010b). *l*_1_/2 regularization. Sci. China Inform. Sci. 53, 1159–1169. 10.1007/s11432-010-0090-0

[B48] Zhang D. Shen D. Initiative A. D. N.. (2012). Multi-modal multi-task learning for joint prediction of multiple regression and classification variables in Alzheimer's disease. NeuroImage 59, 895–907. 10.1016/j.neuroimage.2011.09.069 21992749 PMC3230721

[B49] Zhang J. Zhao S. Huang W. Hu S. (2017). Brain effective connectivity analysis from eeg for positive and negative emotion, in International Conference on Neural Information Processing. (Springer), 851–857.

[B50] Zheng W.-L. Lu B.-L. (2015). Investigating critical frequency bands and channels for eeg-based emotion recognition with deep neural networks. IEEE Trans. Auton. Mental Dev. 7, 162–175. 10.1109/TAMD.2015.2431497

[B51] Zheng W.-L. Zhu J.-Y. Lu B.-L. (2017). Identifying stable patterns over time for emotion recognition from eeg. IEEE Trans. Affect. Comput. 10, 417–429. 10.1109/TAFFC.2017.2712143

[B52] Zhou J. Liu J. Narayan V. A. Ye J. (2012). Modeling disease progression via fused sparse group lasso, in Proceedings of the 18th ACM SIGKDD International Conference on Knowledge Discovery and Data Mining. 1095–1103. 10.1145/2339530.2339702 PMC4191837 25309808

[B53] Zhou Q. (2018). Multi-layer affective computing model based on emotional psychology. Electr. Commerce Res. 18, 109–124. 10.1007/s10660-017-9265-8

[B54] Zhu X. Suk H.-I. Shen D. (2014). A novel matrix-similarity based loss function for joint regression and classification in ad diagnosis. NeuroImage 100, 91–105. 10.1016/j.neuroimage.2014.05.078 24911377 PMC4138265

[B55] Zong-Ben X. Hai-Liang G. Yao W. Zhang H. (2012). Representative of *l*_1_/2 regularization among lq (0 < q ≤ 1) regularizations: an experimental study based on phase diagram. Acta Autom. Sinica 38, 1225–1228. 10.1016/S1874-1029(11)60293-0

