# Supplementary material for: Sparse Granger Causality Analysis Model Based on Sensors Correlation for Emotion Recognition Classification in Electroencephalography
Source: Front Comput Neurosci. 2021 Jul 29;15:684373. doi: 10.3389/fncom.2021.684373 (PMC8358835; doi:10.3389/fncom.2021.684373)
Supplement: Supplementary file 1 [file Data_Sheet_1.docx]

| Method | Feature extraction method | Neutral | | Positive | | Negative | |
| --- | --- | --- | --- | --- | --- | --- | --- |
|  |  | Precision | Recall | Precision | Recall | Precision | Recall |
| SVM | Original | 0.6200±0.0354 | 0.7381±0.0363 | 0.6923±0.0371 | 0.6±0.0328 | 0.6047±0.0344 | 0.5778±0.0347 |
|  | LASSO-GA | 0.7447±0.0265 | 0.7778±0.0242 | 0.8444±0.0244 | 0.8444±0.0250 | 0.8000±0.0255 | 0.7619±0.0251 |
|  | LAPPS | 0.8222±0.0147 | 0.8605±0.0161 | 0.9000±0.0161 | 0.8524±0.0138 | 0.8108±0.0159 | 0.7895±0.0166 |
|  | SC-SGA | 0.8470±0.0105 | 0.8703±0.0099 | 0.9011±0.0112 | 0.8625±0.0108 | 0.8454±0.0117 | 0.8203±0.0096 |
| Logistics Regression | Original | 0.7209±0.0358 | 0.7381±0.0362 | 0.7317±0.0345 | 0.6667±0.0365 | 0.6667±0.0364 | 0.7111±0.0345 |
|  | LASSO-GA | 0.7579±0.0264 | 0.7576±0.0227 | 0.8776±0.0258 | 0.86±0.0248 | 0.8444±0.0251 | 0.7755±0.0260 |
|  | LAPPS | 0.8571±0.0137 | 0.7826±0.0135 | 0.9167±0.0148 | 0.8684±0.0147 | 0.8478±0.0153 | 0.875±0.0154 |
|  | SC-SGA | 0.8889±0.0106 | 0.7912±0.0107 | 0.9266±0.0096 | 0.8747±0.0095 | 0.8634±0.0114 | 0.8916±0.0111 |

TABLE I

PRECISION AND RECALL RESULTS FOR SVM and L_1_ IN GAMMA BAND OF SEED DATASET.

TABLE II

PRECISION AND RECALL RESULTS FOR SVM and L_1_ IN Combined BAND OF SEED DATASET.

| Method | Feature extraction method | Neutral | | Positive | | Negative | |
| --- | --- | --- | --- | --- | --- | --- | --- |
|  |  | Precision | Recall | Precision | Recall | Precision | Recall |
| SVM | Original | 0.7805±0.0344 | 0.6809±0.0335 | 0.7727±0.0365 | 0.8718±0.0327 | 0.7660±0.0339 | 0.7826±0.0327 |
|  | LASSO-GA | 0.8085±0.0278 | 0.8085±0.0236 | 0.8333±0.0278 | 0.8974±0.0264 | 0.8140±0.0224 | 0.7609±0.0236 |
|  | LAPPS | 0.7391±0.0156 | 0.8500±0.0147 | 0.8368±0.0168 | 0.9216±0.0171 | 0.8182±0.0150 | 0.6585±0.0156 |
|  | SC-SGA | 0.8156±0.0112 | 0.8796±0.0110 | 0.8413±0.0108 | 0.9345±0.0101 | 0.8413±0.0107 | 0.8057±0.0106 |
| Logistics regression | Original | 0.7872±0.0356 | 0.7872±0.0343 | 0.8372±0.0336 | 0.8231±0.0376 | 0.8095±0.0342 | 0.7391±0.0344 |
|  | LASSO-GA | 0.7660±0.0265 | 0.7727±0.0276 | 0.8723±0.0234 | 0.8333±0.0256 | 0.7826±0.0226 | 0.8182±0.0231 |
|  | LAPPS | 0.8049±0.0171 | 0.825±0.0164 | 0.8462±0.0152 | 0.8465±0.0134 | 0.7674±0.0147 | 0.8049±0.0158 |
|  | SC-SGA | 0.8356±0.0120 | 0.8578±0.0116 | 0.8978±0.0106 | 0.8869±0.0102 | 0.8256±0.098 | 0.8413±0.0104 |


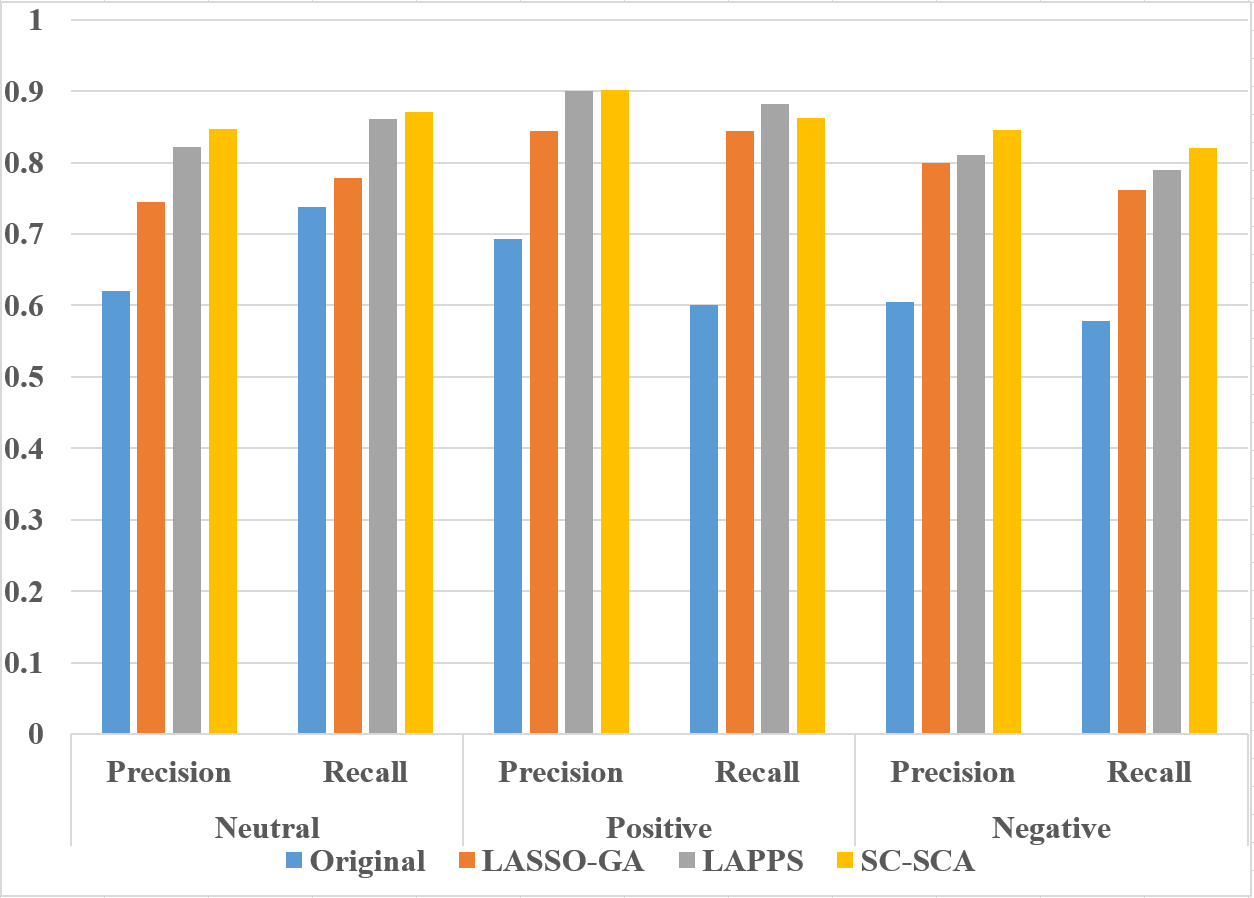


Fig 1. Histogram of precision and recall of the four models using SVM for the Gamma band of the SEED dataset.


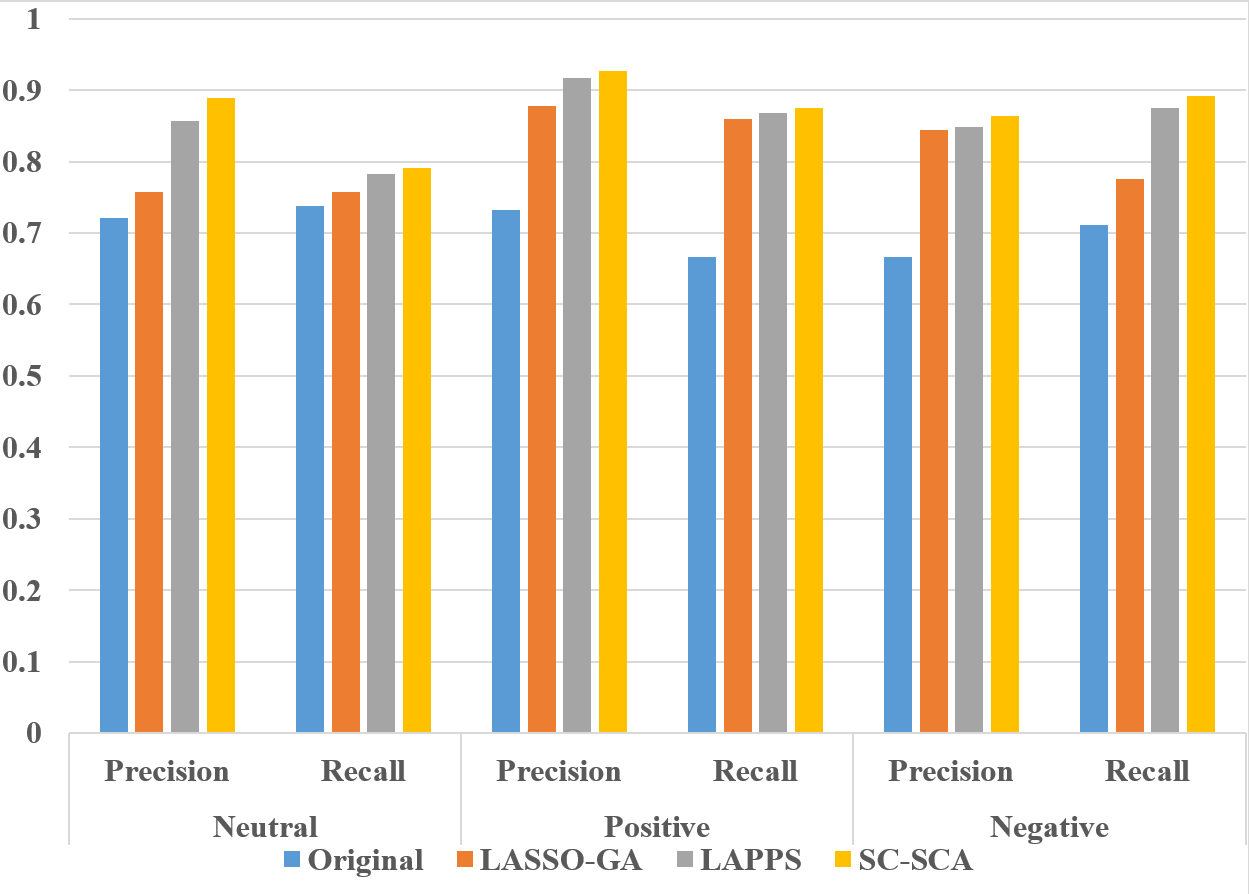


Fig 2. Histogram of precision and recall of the four models using the sparse logistic regressions with L_1_ penalty for the Gamma band of the SEED dataset.


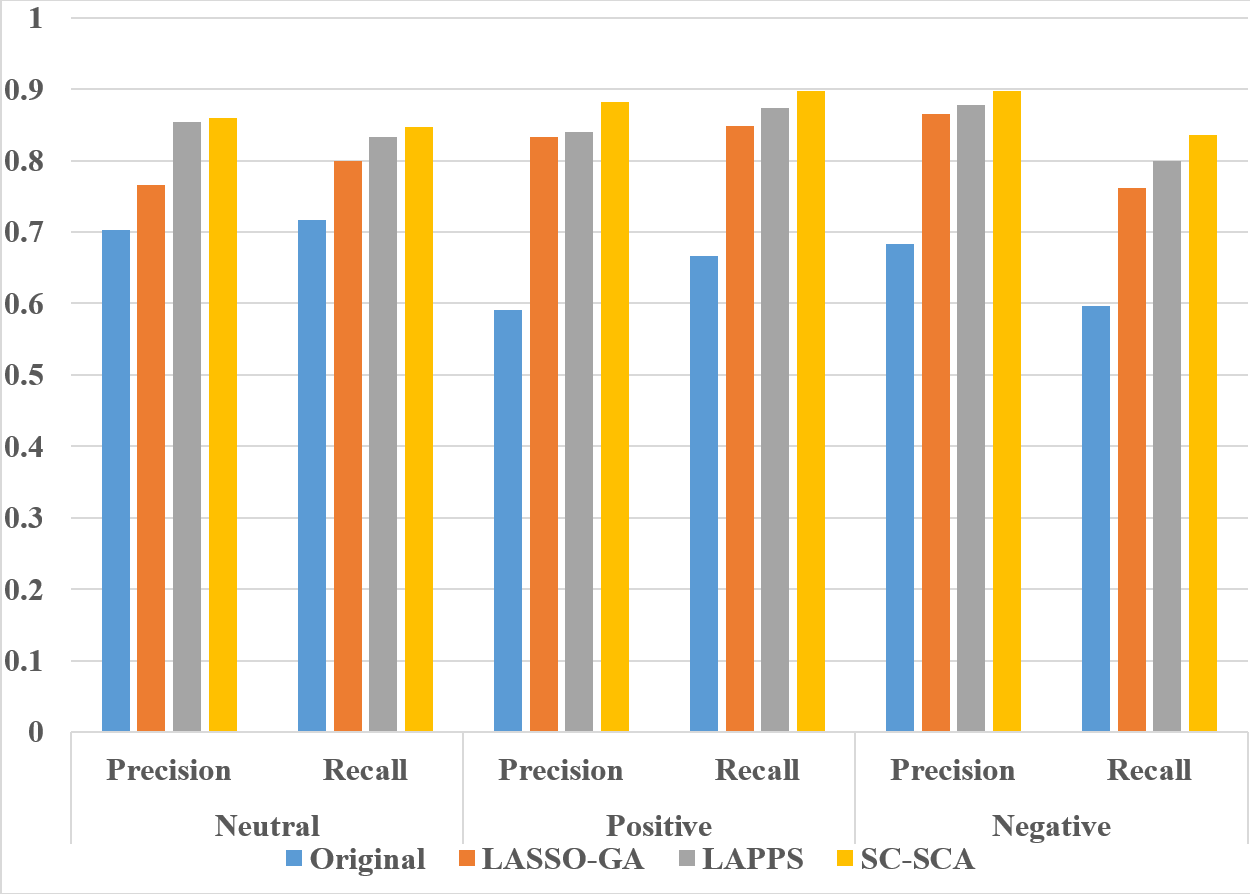


Fig 3. Histogram of precision and recall of the four models using Ridge Regressions for the Gamma band of the SEED dataset.


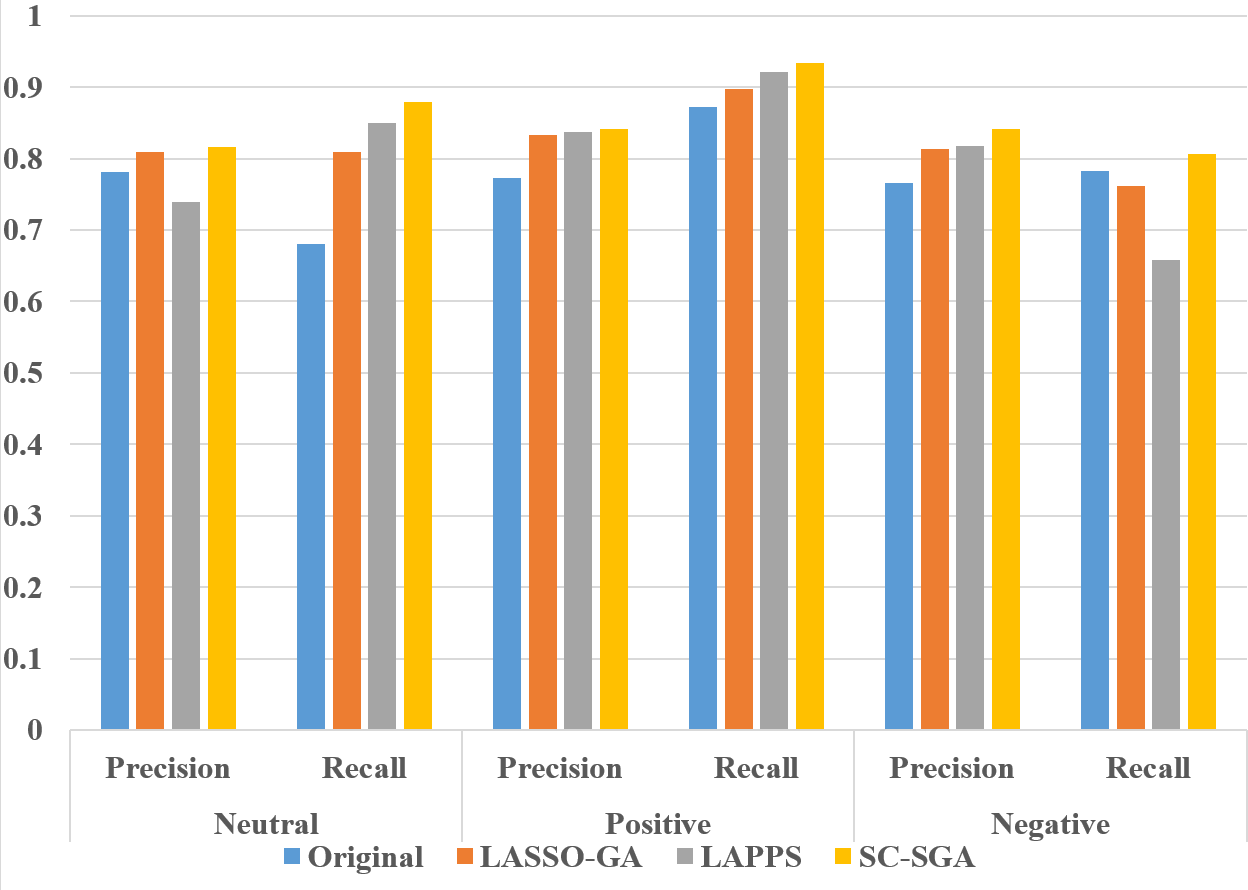


Fig 4. Histogram of precision and recall of the four models using SVM for the Combined band of the SEED dataset.


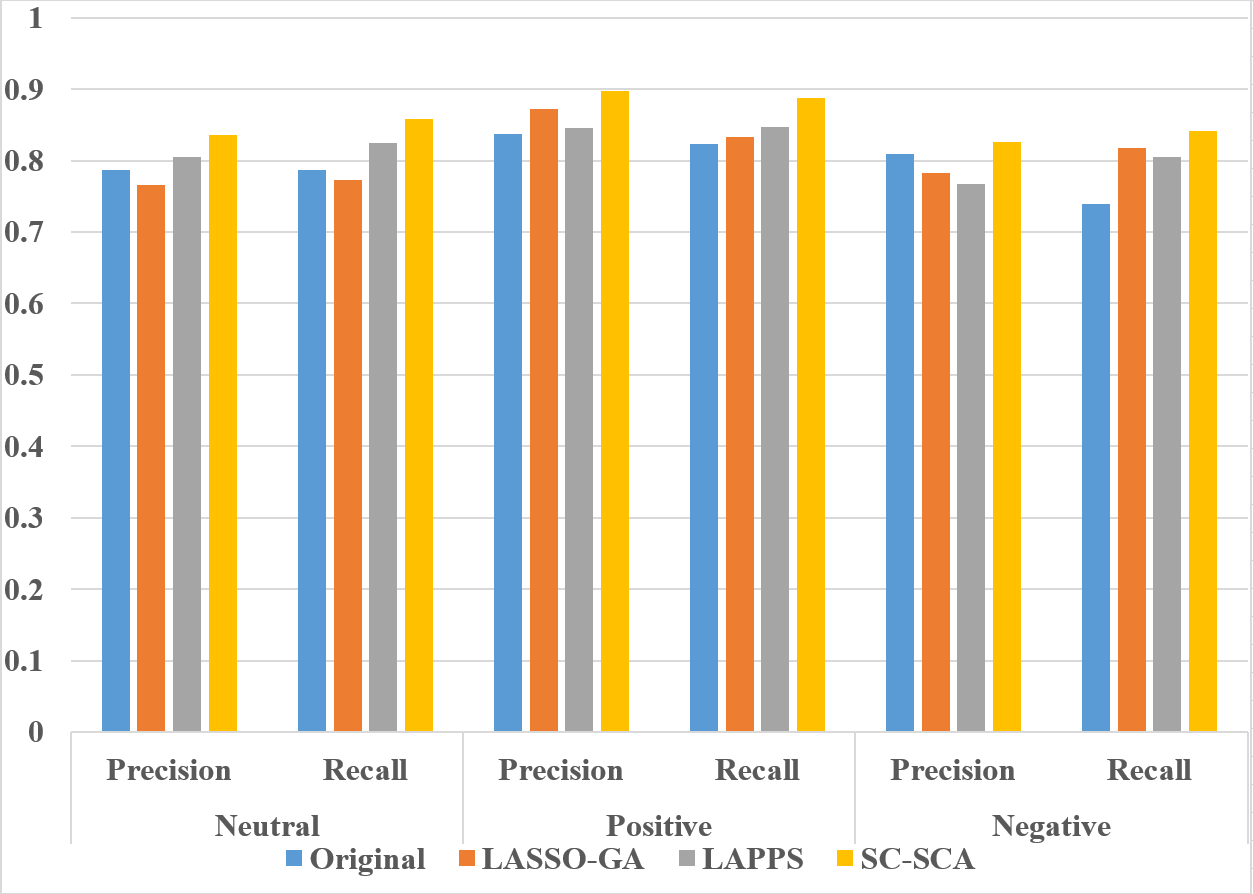


Fig 5. Histogram of precision and recall of the four models using the sparse logistic regressions with L_1_ penalty for the Combined band of the SEED dataset.


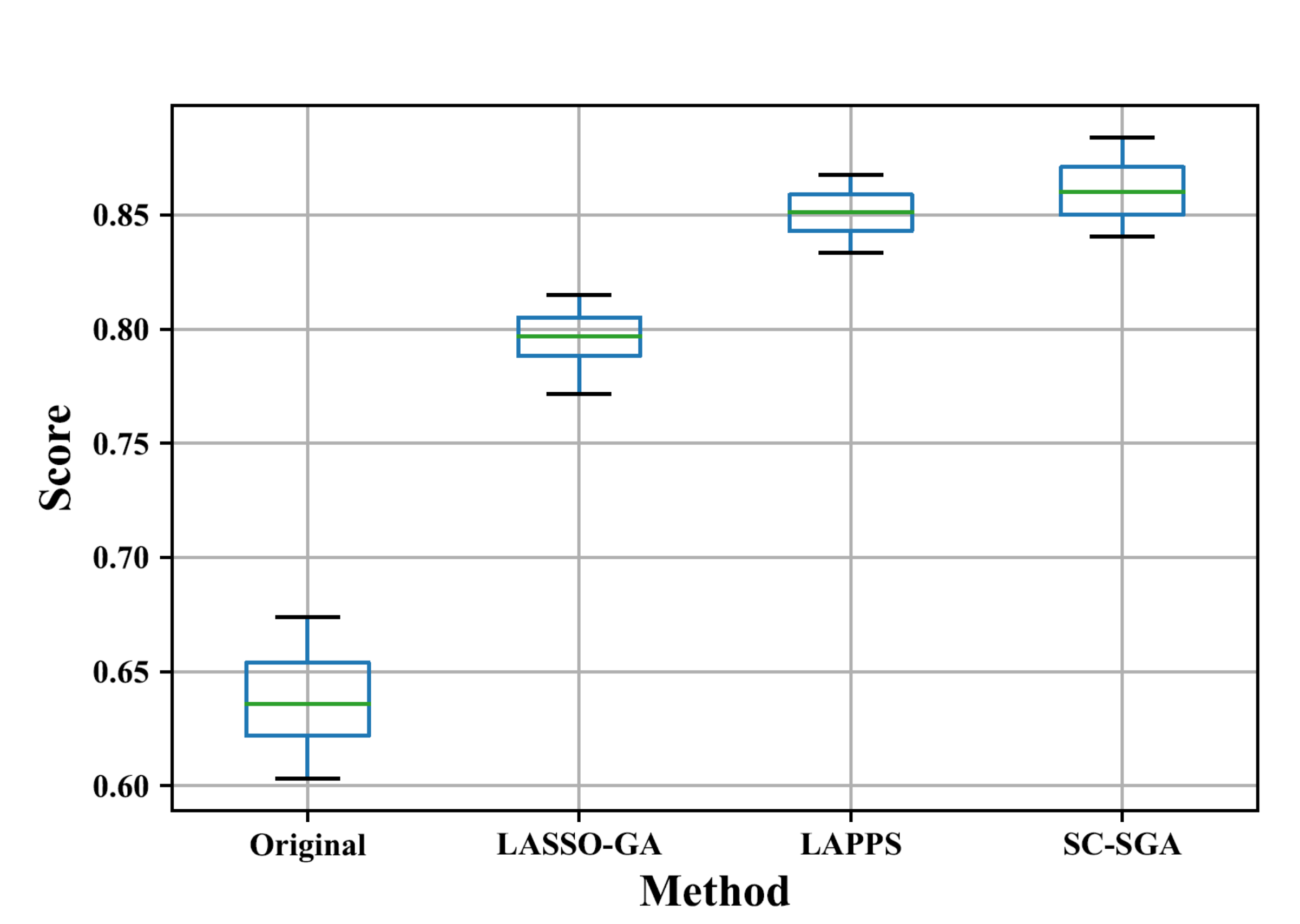


Fig 6. Box plot obtained by using SVM under the four models of the Gamma band of the SEED dataset.


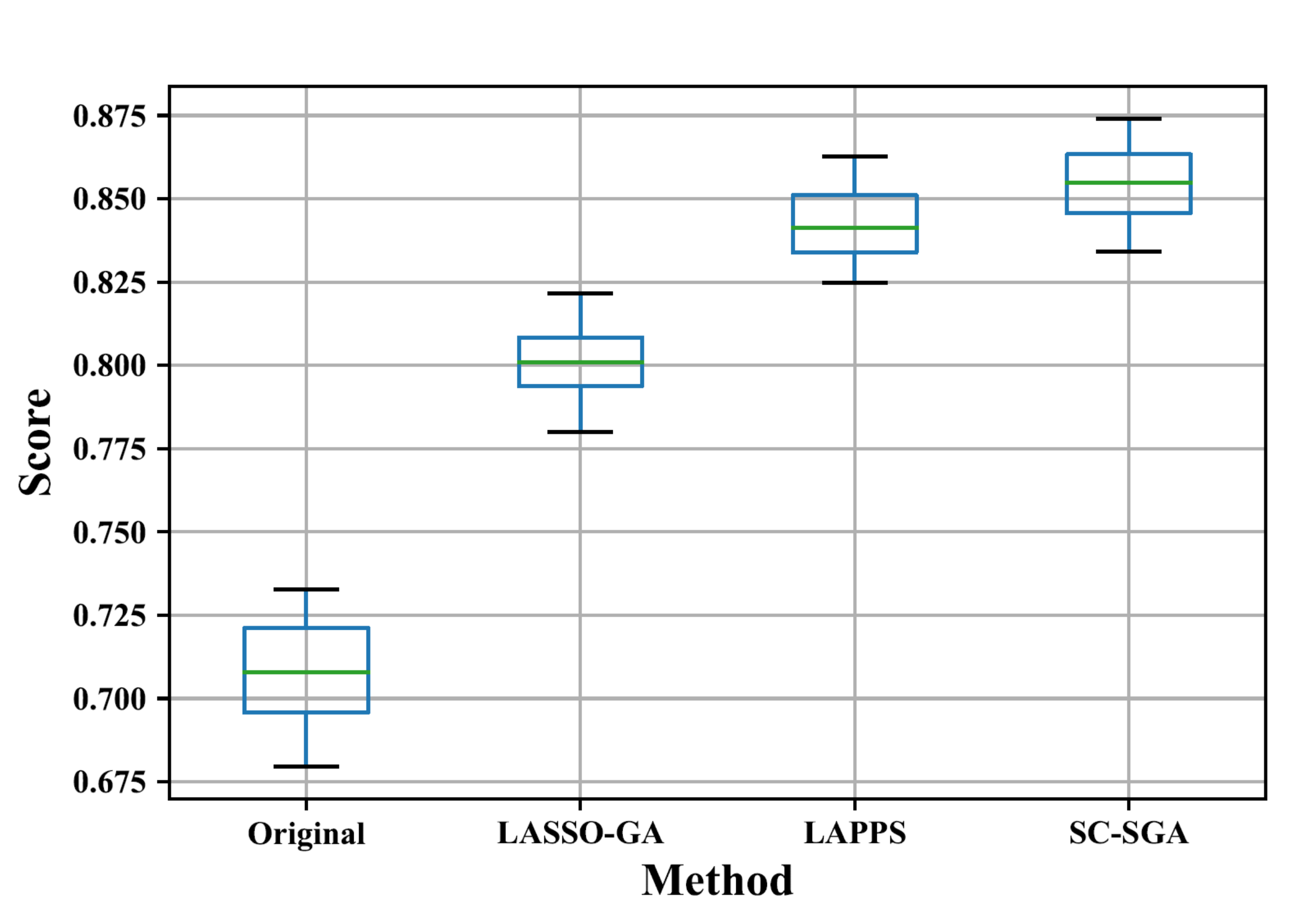


Fig 7. Box plot obtained by using the sparse logistic regressions with L_1_ penalty under the four models of the Gamma band of the SEED dataset.


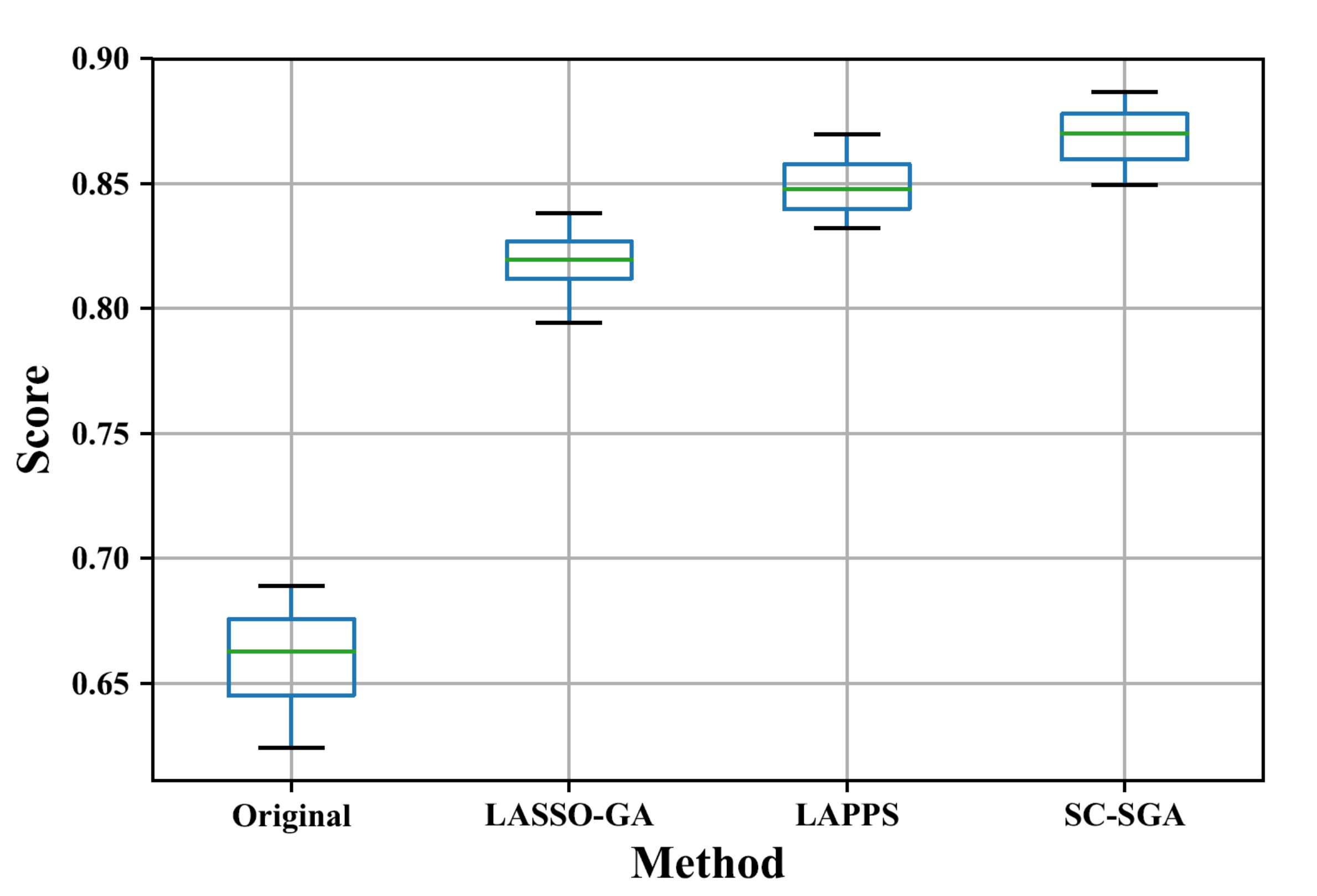


Fig 8. Box plot obtained by using Ridge Regressions under the four models of the Gamma band of the SEED dataset.


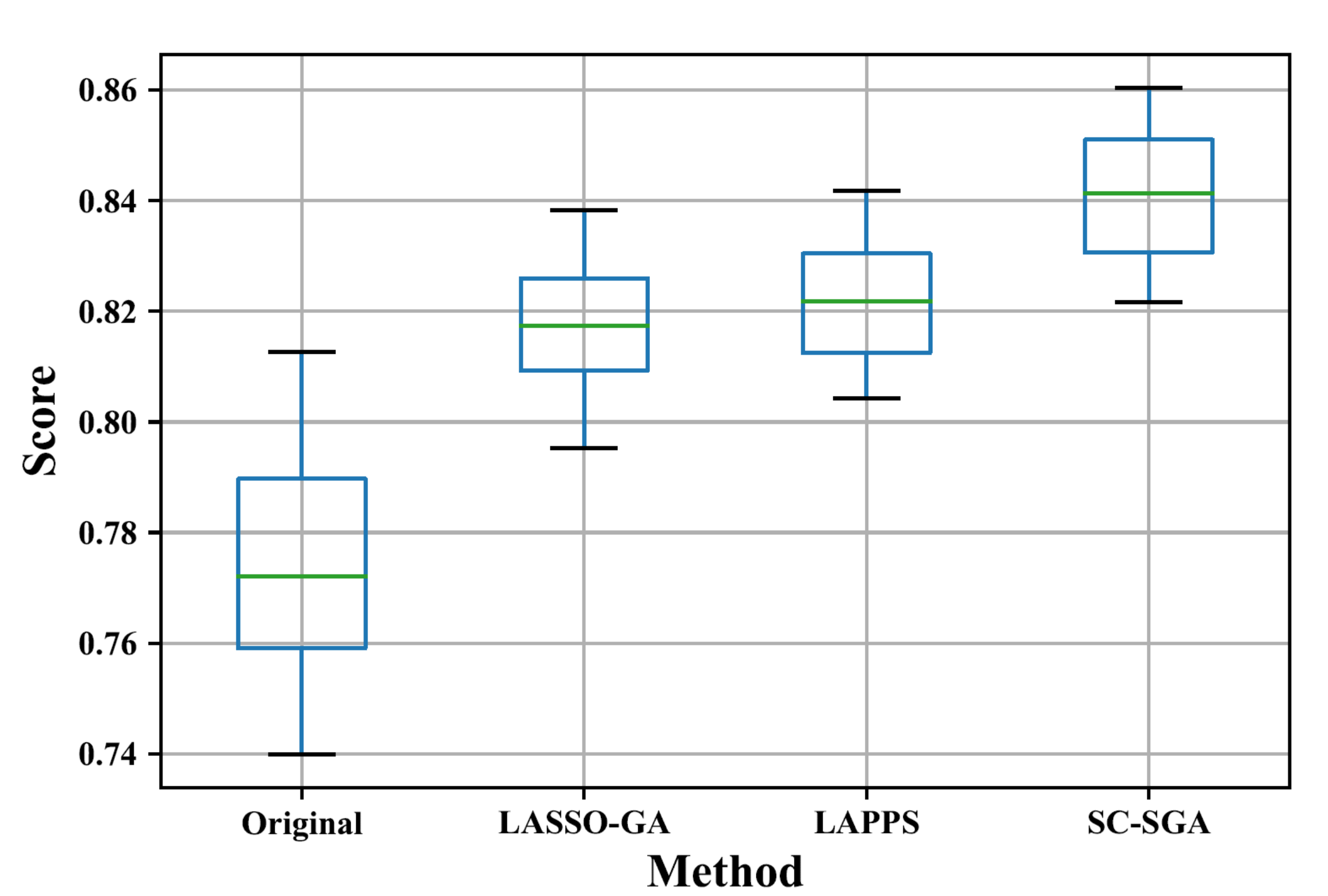


Fig 9. Box plot obtained by using SVM under the four models of the Combined band of the SEED dataset.


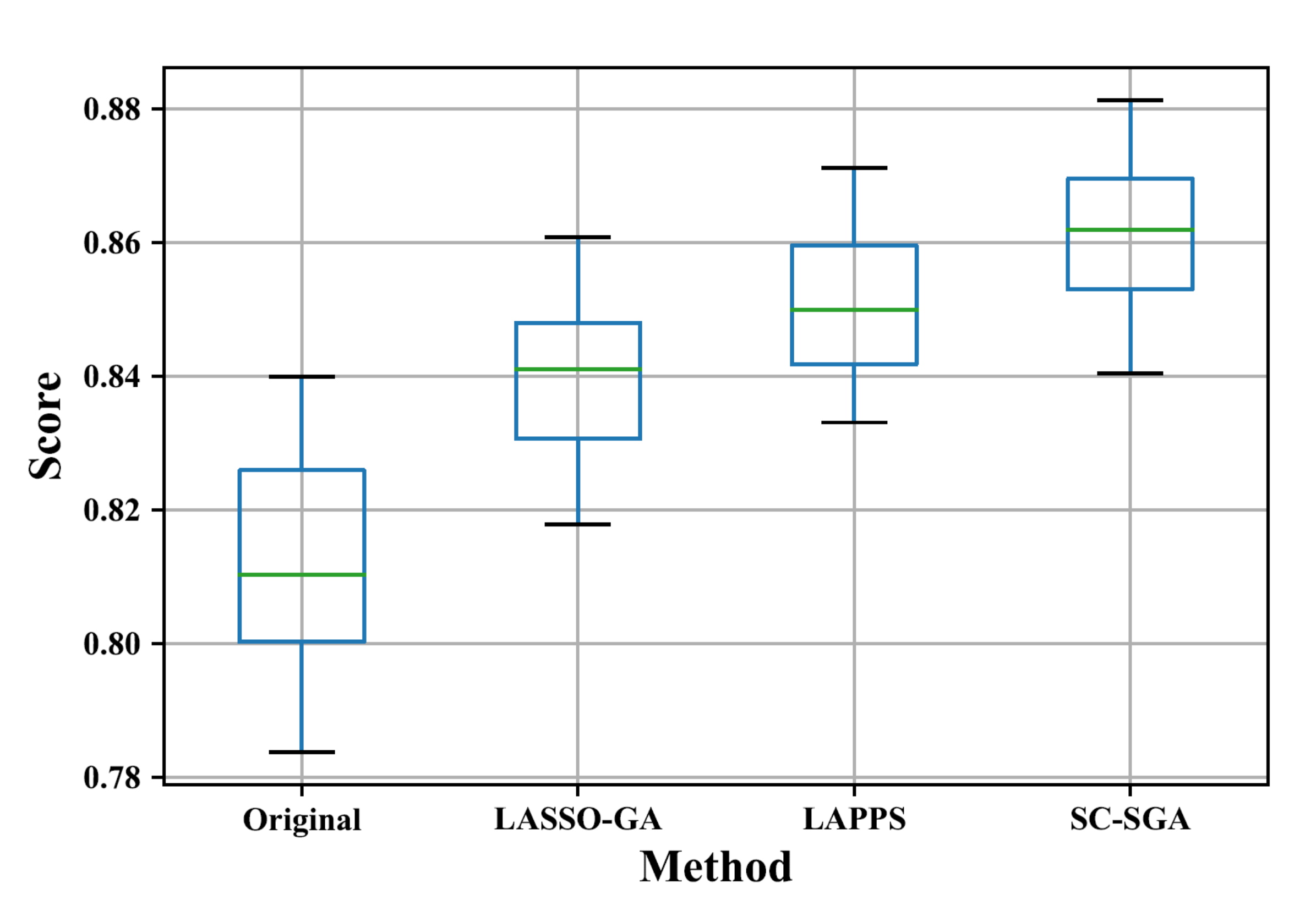


Fig 10. Box plot obtained by using the sparse logistic regressions with L_1_ penalty under the four models of the Combined band of the SEED dataset.


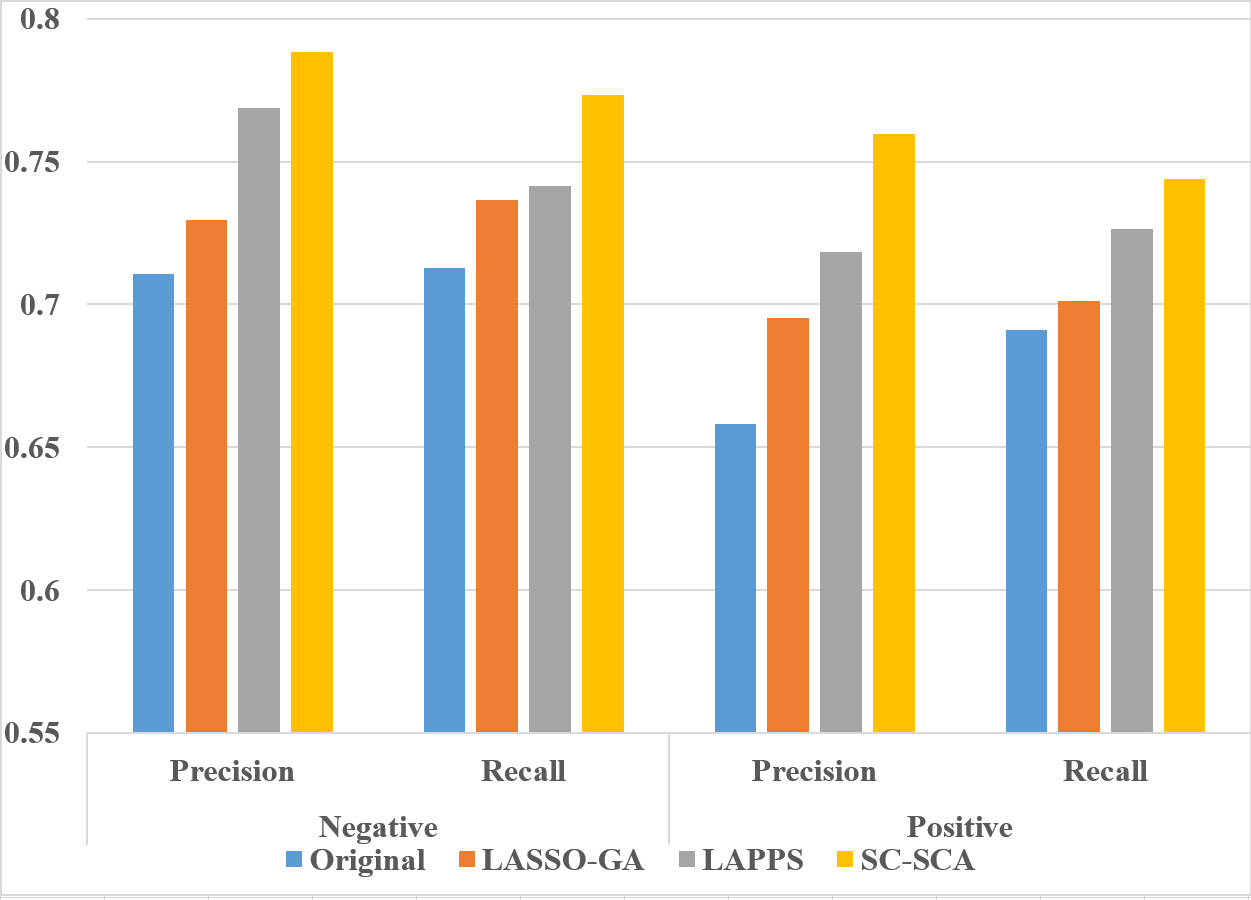


Fig 11. Histogram of precision and recall of the four models using SVM for the DEAP dataset.


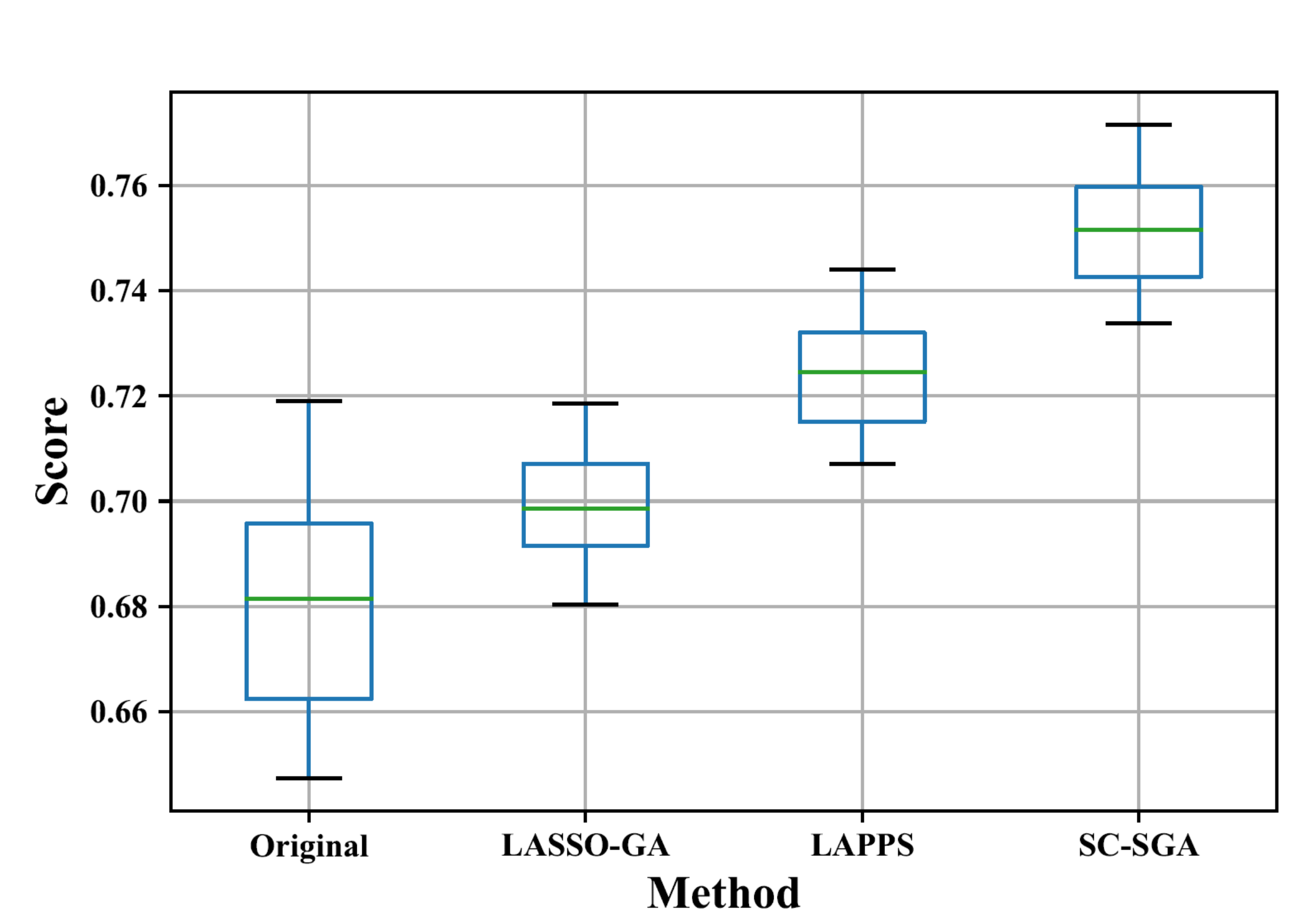


Fig 12. Box plot obtained by using SVM under the four models of the DEAP dataset.


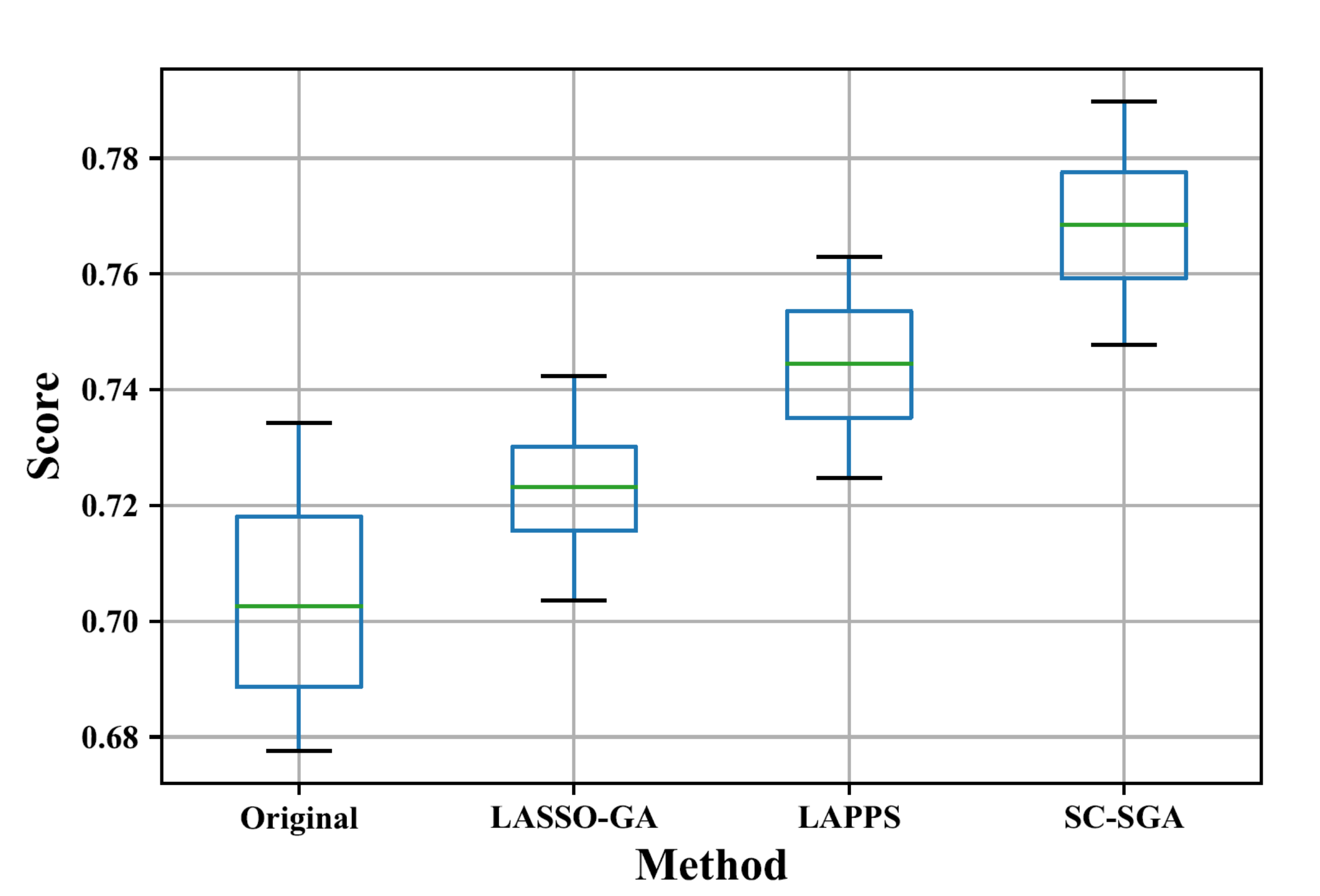


Fig 13. Box plot obtained by using the sparse logistic regressions with L_1_ penalty under the four models of the DEAP dataset.


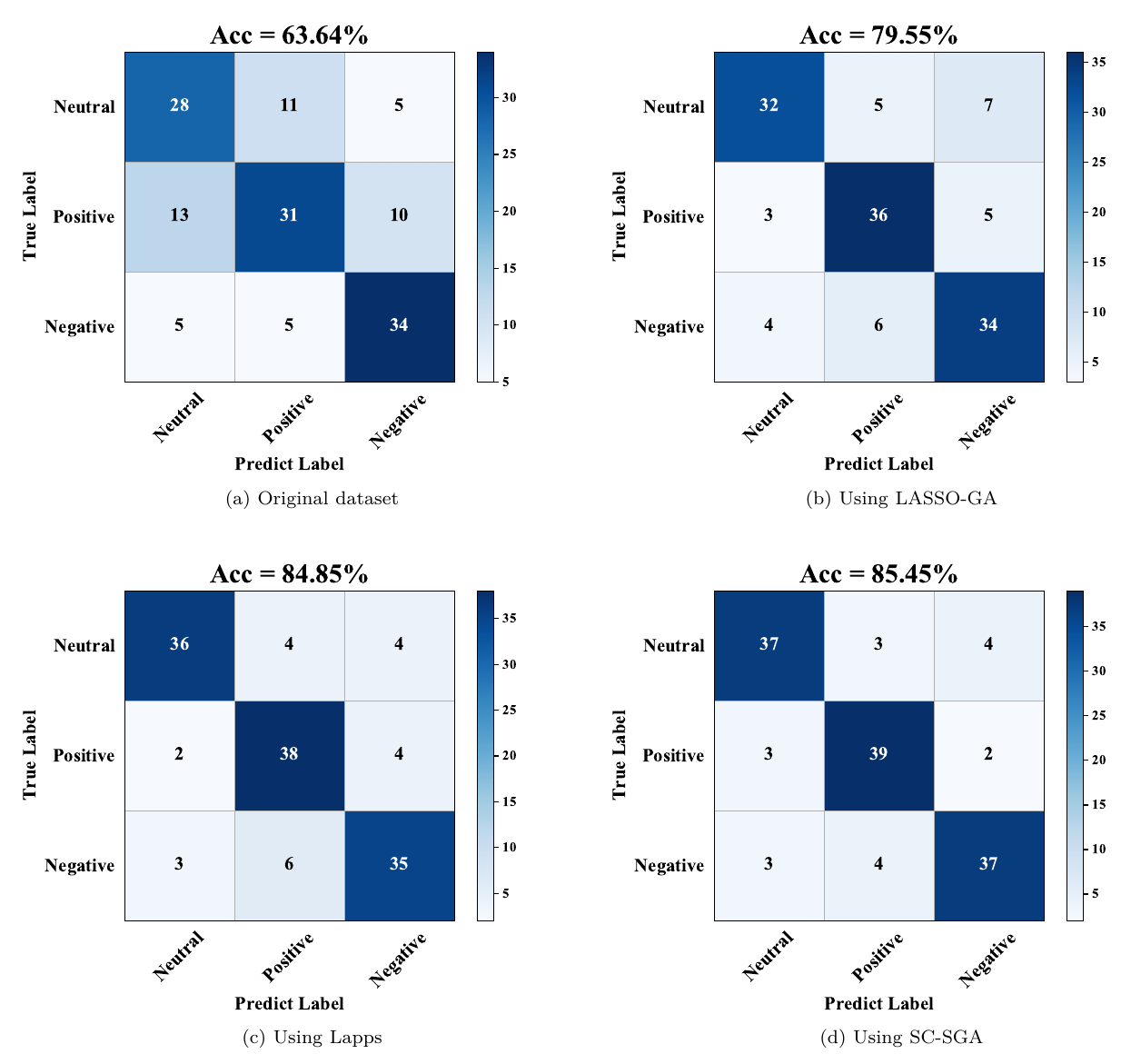


Fig 14. The confusion matrix obtained by using SVM under the processing of the four models for the gamma band of the SEED dataset.


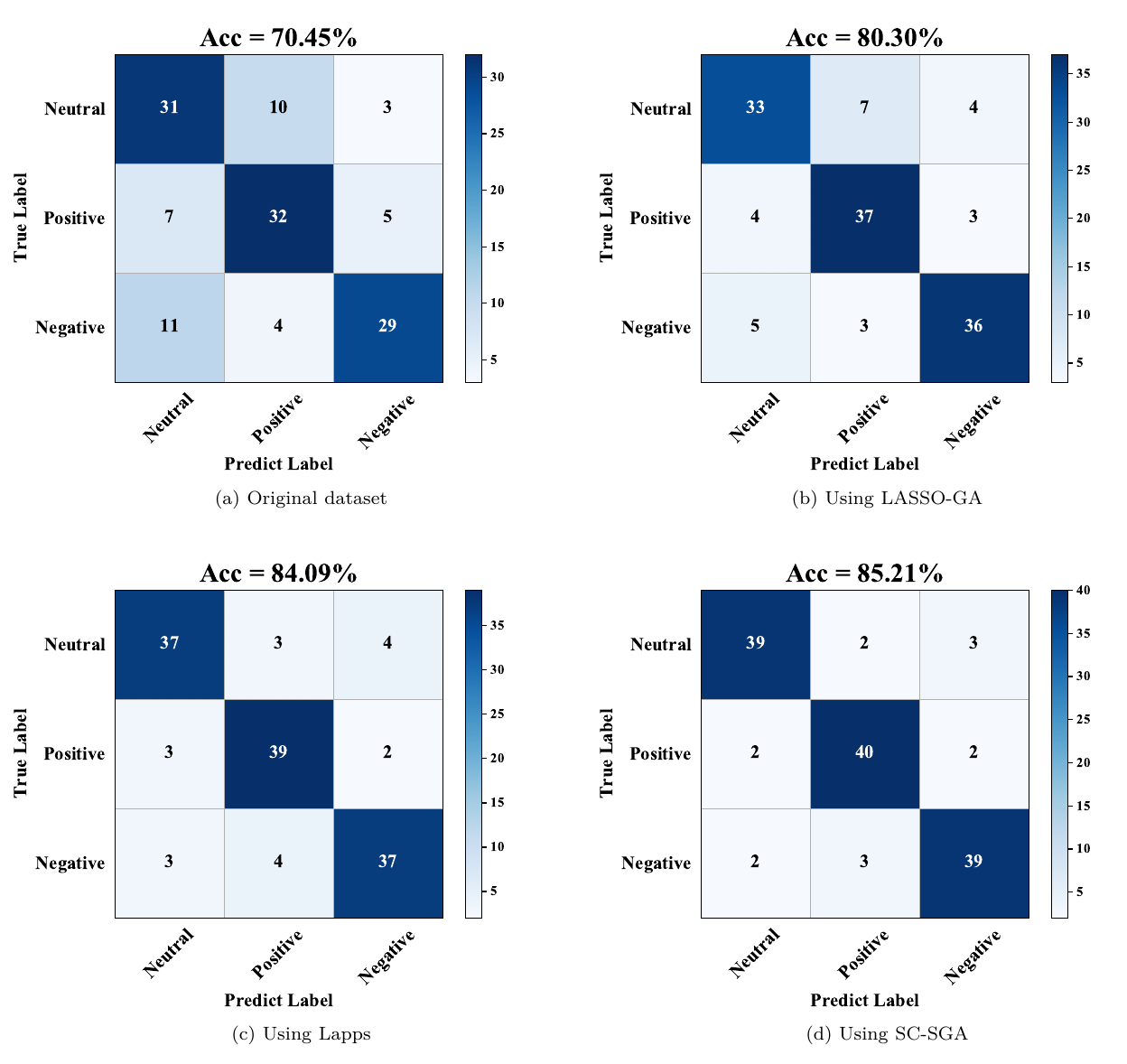


Fig 15. The confusion matrix obtained by using sparse logistic regressions with L_1_ penalty under the processing of the four models for the gamma band of the SEED dataset.


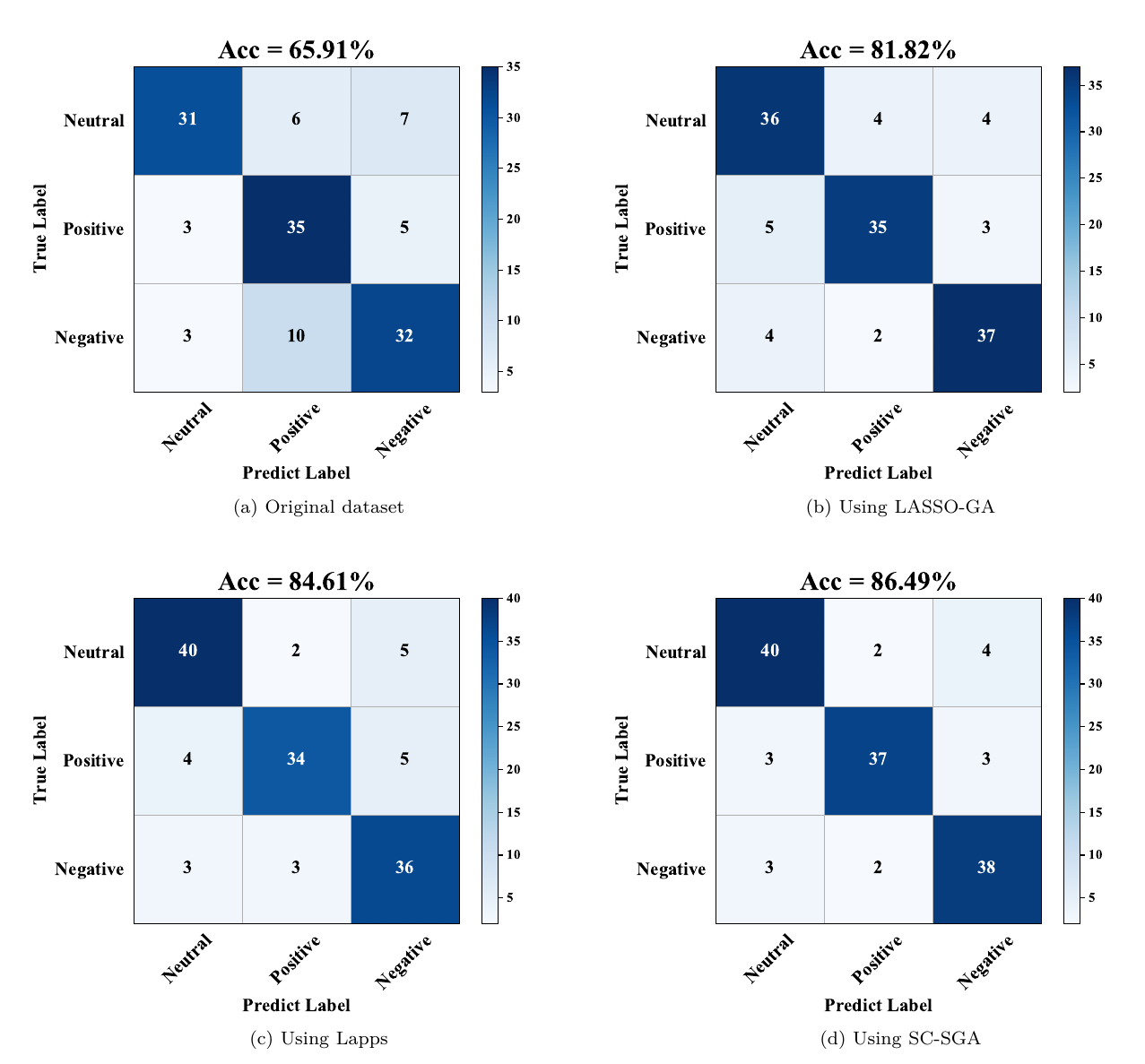


Fig 16. The confusion matrix obtained by using ridge regression under the processing of the four models for the gamma band of the SEED dataset.


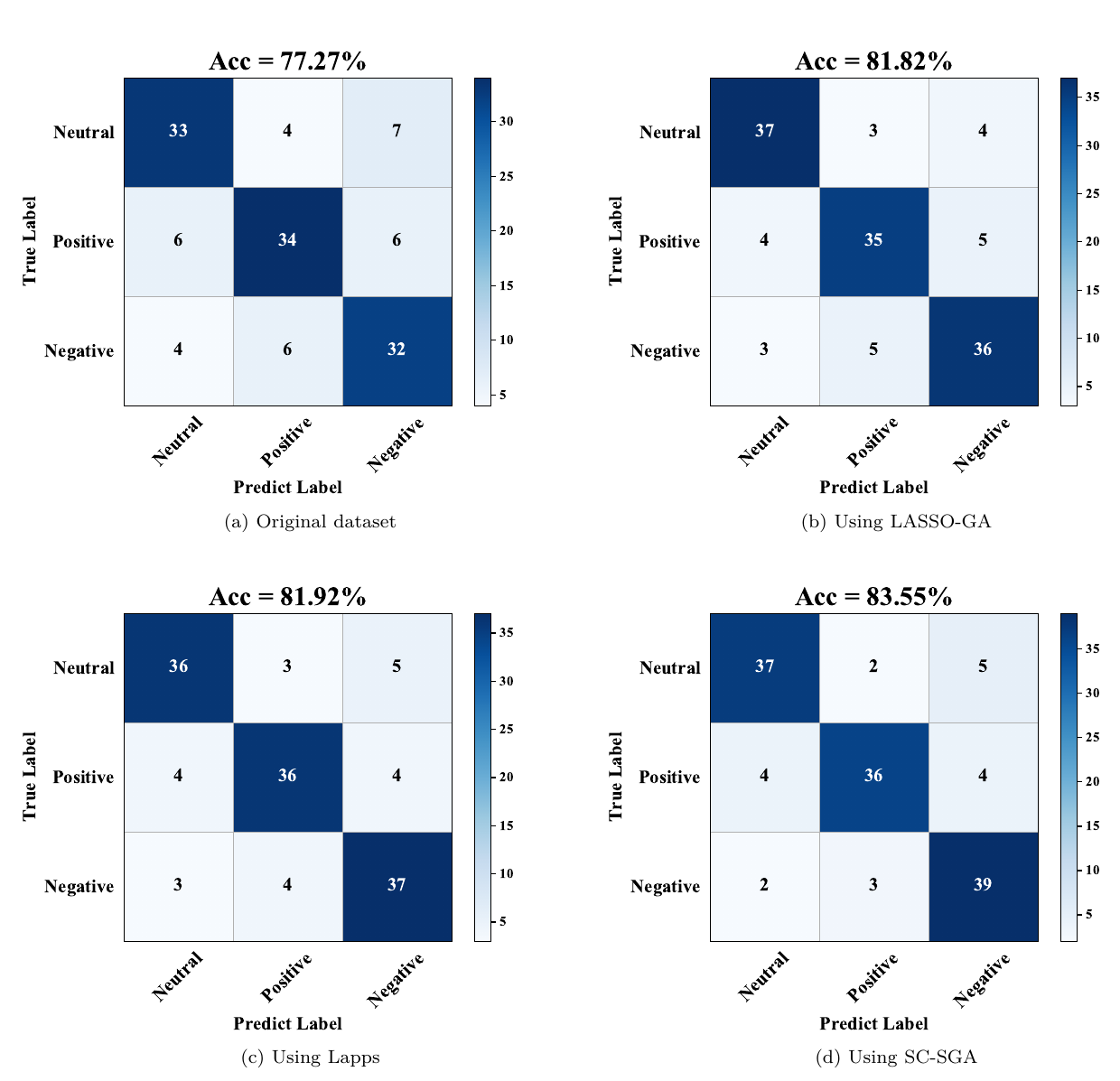


Fig 17. The confusion matrix obtained by using SVM under the processing of the four models for the combined band of the SEED dataset.


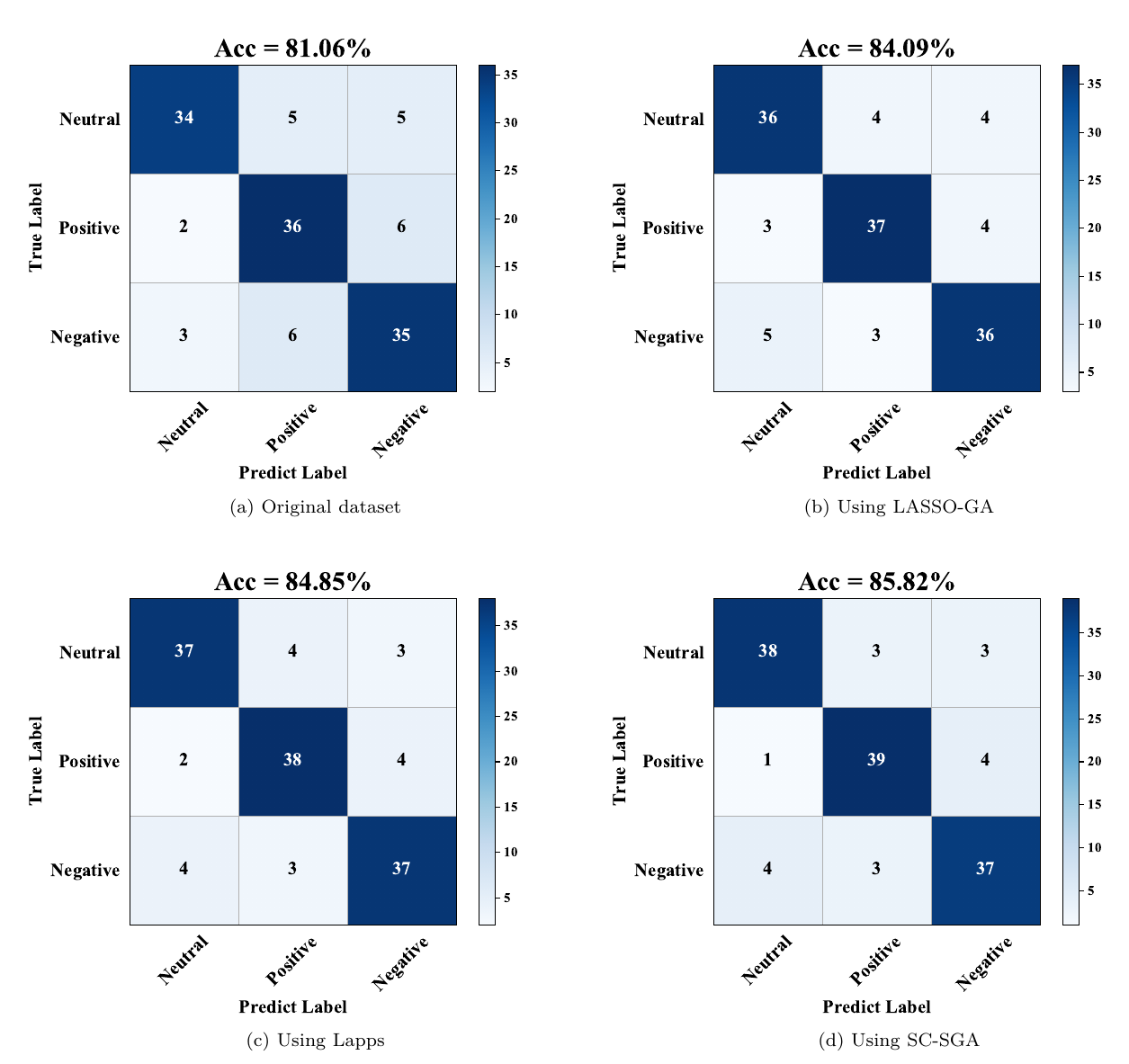


Fig 17. The confusion matrix obtained by using sparse logistic regressions with L_1_ penalty under the processing of the four models for the combined band of the SEED dataset.

TABLE III

PRECISION AND RECALL RESULTS FOR SVM and L_1_ IN DEAP DATASET.

| Method | Feature extraction method | Positive | | Negative | |
| --- | --- | --- | --- | --- | --- |
|  |  | Precision | Recall | Precision | Recall |
| SVM | GA | 0.7108±0.0325 | 0.7128±0.0328 | 0.6582±0.0341 | 0.6912±0.0336 |
|  | LASSO-GA | 0.7297±0.0270 | 0.7367±0.0264 | 0.6952±0.0220 | 0.7013±0.0232 |
|  | LAPPS | 0.7688±0.0180 | 0.7416±0.01860 | 0.7182±0.01750 | 0.7265±0.0174 |
|  | SC-SGA | 0.7884±0.0115 | 0.7733±0.0109 | 0.7596±0.0103 | 0.7438±0.0106 |
| Logistics regression | GA | 0.7143±0.0345 | 0.7000±0.0347 | 0.6815±0.0358 | 0.7183±0.0362 |
|  | LASSO-GA | 0.7218±0.0276 | 0.7158±0.0264 | 0.6821±0.0251 | 0.7297±0.0246 |
|  | LAPPS | 0.7442±0.0186 | 0.7465±0.0175 | 0.7358±0.0163 | 0.7546±0.0169 |
|  | SC-SGA | 0.7532±0.0102 | 0.7689±0.0096 | 0.7615±0.0112 | 0.7800±0.0116 |
